# Analytical Validation and Preliminary Diagnostic Performance Evaluation of GenoPATHX™ Multiplex qPCR for Quantitative Detection of Key *Salmonella* Serovars in Poultry Matrices

**DOI:** 10.3390/pathogens15080791

**Published:** 2026-07-25

**Authors:** Rejoice Nyarku, Emmanuel Kuufire, Viona Osei, Kingsley E. Bentum, Asmaa Elrefaey, Emmanuel Piiru, Tyric James, Yilkal Woube, Temesgen Samuel, Woubit Abebe

**Affiliations:** Center for Food Animal Health, Food Safety and Defense, Department of Pathobiology, College of Veterinary Medicine, Tuskegee University, Tuskegee, AL 36088, USA; rnyarku8794@tuskegee.edu (R.N.); ekuufire9436@tuskegee.edu (E.K.); vosei3882@tuskegee.edu (V.O.); kbentum@tuskegee.edu (K.E.B.); aelrefaey3754@tuskegee.edu (A.E.); epiiru3315@tuskegee.edu (E.P.); tjames2981@tuskegee.edu (T.J.); ywoube@tuskegee.edu (Y.W.); tsamuel@tuskegee.edu (T.S.)

**Keywords:** *Salmonella enterica*, multiplex qPCR, quantitative detection, poultry matrices, diagnostic performance evaluation

## Abstract

Rapid detection and quantification of epidemiologically important *Salmonella enterica* serovars are critical for poultry surveillance, food safety monitoring, and risk-based intervention strategies. This study performed comprehensive analytical validation together with a preliminary field-based diagnostic performance evaluation of GenoPATHX™, a multiplex probe-based qPCR platform designed for the simultaneous detection and quantification of priority *Salmonella* serovars in poultry-associated matrices. The platform consists of two multiplex panels, designated the Chicken Key Performance Indicator (CKPI) and Turkey Key Performance Indicator (TKPI), each designed to detect priority poultry-associated *Salmonella* serovars together with a genus-level *S. enterica* marker. Analytical performance was evaluated for amplification efficiency, linearity, limit of detection (LoD_95_), limit of quantification (LoQ), repeatability, intermediate precision, analytical specificity (inclusivity/exclusivity), robustness, matrix effects, and performance in artificially inoculated matrices. Diagnostic performance was further assessed using naturally contaminated poultry environmental samples. The assay demonstrated robust amplification performance in both singleplex and multiplex formats, with high linearity (R^2^ = 0.987–0.999) and LoD_95_ values ranging from 60 to 545 genome equivalents per reaction. Complete analytical inclusivity and high exclusivity were achieved for the evaluated isolate panel. In field samples, the direct GenoPATHX™ workflow demonstrated 81.0% sensitivity, 91.3% specificity, and substantial agreement with the USDA-FSIS reference culture method (κ = 0.73). Overall, GenoPATHX™ exhibited robust analytical performance and enabled rapid, same-day quantitative detection of priority *Salmonella* serovars in poultry-associated matrices, supporting its application for poultry surveillance and food safety monitoring.

## 1. Introduction

*Salmonella enterica* remains a major global public health concern, with poultry and poultry products serving as a primary source of human exposure [1]. Estimates from the World Health Organization (WHO) Foodborne Disease Burden Epidemiology Reference Group (FERG, 2007–2015) indicate that non-typhoidal *Salmonella enterica* and invasive non-typhoidal *Salmonella enterica* account for approximately 4.38 million (3.24–7.18) and 3.9 million (2.4–5.8) disability-adjusted life years (DALYs), respectively [2]. In the United States, the economic burden on the poultry industry is substantial, with flock losses and production disruptions estimated to cost between $64 million and $114 million annually [3]. Poultry frequently harbor *Salmonella* asymptomatically, continuously shedding the organism into the production environment [4]. The persistence of *Salmonella* within poultry houses between production cycles further complicates control efforts [5], underscoring the importance of continuous surveillance and rapid detection to interrupt contamination cycles [6,7,8]. Human infection is commonly associated with the consumption of raw or undercooked poultry products, emphasizing the need for effective farm-to-fork control strategies [9].

The Centers for Disease Control and Prevention (CDC) estimates that *Salmonella* causes approximately 1.35 million infections annually in the United States [10]. Although more than 2600 *Salmonella* serovars have been identified, relatively few account for the majority of human infections and poultry-associated outbreaks [11,12,13,14]. Among these, *S.* Enteritidis, *S.* Typhimurium, and monophasic *S.* Typhimurium (1,4,[5],12:i:-) are consistently recognized as leading causes of foodborne illness in both the United States and the European Union [15,16]. Likewise, poultry surveillance programs have identified *S.* Enteritidis, *S.* Typhimurium, monophasic *S.* Typhimurium, *S.* Hadar, and *S.* Muenchen as priority serovars because of their frequent association with poultry products and human disease [17]. Consequently, rapid and accurate detection of these priority serovars is essential for targeted surveillance, outbreak investigations, and risk-based food safety interventions.

Increasing evidence suggests that targeted control strategies focusing on high-risk serovars associated with severe human disease are more effective than broad-spectrum approaches [14]. Consequently, industry, academic, and regulatory efforts have increasingly shifted toward the rapid identification and monitoring of these priority serovars [18,19,20]. Despite remaining the regulatory reference standard, culture-based methods such as the USDA-FSIS Microbiology Laboratory Guidebook (MLG 4.15) are labor-intensive and typically require several days to generate confirmed results [21]. Although quantitative PCR (qPCR) has substantially improved detection speed and analytical sensitivity, many currently available assays have limited multiplexing capacity and cannot simultaneously differentiate closely related epidemiologically important serovars within a single reaction [22,23]. Furthermore, relatively few platforms support direct quantitative detection in complex poultry-associated matrices without extended enrichment.

To address these limitations, we evaluated GenoPATHX™, a proprietary multiplex probe-based qPCR platform developed for the rapid detection and quantification of epidemiologically important *Salmonella enterica* serovars associated with poultry production systems. The platform comprises two multiplex panels: the Chicken Key Performance Indicator (CKPI), targeting *S.* Typhimurium, *S.* Enteritidis, and monophasic *S.* Typhimurium, and the Turkey Key Performance Indicator (TKPI), targeting *S.* Typhimurium, *S.* Muenchen, and *S.* Hadar. Both panels additionally include a genus-level *S. enterica* target to enable broad *Salmonella* detection.

The analytical specificity of the GenoPATHX™ platform was assessed using a two-stage approach consisting of in silico BLASTn analysis followed by experimental inclusivity and exclusivity testing with target and closely related non-target organisms. Analytical performance was further evaluated in terms of amplification efficiency, linearity, quantitative range, repeatability, intermediate precision, robustness, and performance in complex poultry-associated matrices, including poultry rinsates and ground meat. The effect of short non-selective enrichment on assay sensitivity was also investigated. Finally, preliminary diagnostic performance was evaluated using naturally contaminated poultry environmental samples by comparing direct and enrichment-based workflows with the USDA-FSIS reference culture method, with isolate confirmation by whole-genome sequencing (WGS). Collectively, these studies establish GenoPATHX™ as a rapid multiplex platform for the serovar-specific detection and quantitative assessment of priority *Salmonella* serovars in poultry production systems.

## 2. Materials and Methods

### 2.1. Bacterial Strains and Culture Conditions

*Salmonella enterica* serovars corresponding to the CKPI and TKPI panels were used throughout this study, including *S.* Typhimurium (ATCC 14028), monophasic *S.* Typhimurium (4,[5],12:i:-; FDA CFSAN138996), *S.* Muenchen (FDA CFSAN138716), *S.* Hadar (FDA BEAR033953), and *S.* Enteritidis (FDA BEAR062010). Unless otherwise indicated, all strains were obtained from the U.S. Food and Drug Administration (FDA), with *S.* Typhimurium ATCC 14028 obtained from the American Type Culture Collection (ATCC) (ATCC; Manassas, VA, USA). The isolates were streaked onto Tryptic Soy Agar (Neogen Corporation, Lansing, MI, USA) and incubated at 37 °C for 18–24 h. A single well-isolated colony from each culture was inoculated into 5 mL of Tryptic Soy Broth (Neogen Corporation, Lansing, MI, USA) and incubated under the same conditions to produce overnight cultures for subsequent experiments.

### 2.2. Analytical Validation

#### 2.2.1. *In-Silico* Specificity Analysis

In silico specificity of the GenoPATHX™ primers was evaluated using the National Center for Biotechnology Information (NCBI) Basic Local Alignment Search Tool (BLASTn) version 2.17.0+ (National Center for Biotechnology Information, Bethesda, MD, USA). Reference genome sequences representing the target serovars included in this study were retrieved from the NCBI nucleotide database using the accession numbers provided in Supplementary Table S1. Forward primers, reverse primers, and predicted amplicon sequences were queried individually against the NCBI nucleotide collection (nr/nt) using the Megablast algorithm to identify exact and near-exact sequence matches. A maximum of 100 sequence hits was retrieved for each query.

Specificity was assessed by determining whether each primer pair and its corresponding predicted amplicon sequence aligned exclusively with the intended *Salmonella enterica* serovar or species target while minimizing homology with non-target *Salmonella* serovars and non-*Salmonella* organisms. Amplicon specificity was further verified by confirming that the predicted PCR products did not produce significant matches to unintended serovars or non-target organisms within the NCBI database.

#### 2.2.2. Amplification Efficiency, Linearity and Linear Dynamic Range

Genomic DNA (75 ng/µL) from each *Salmonella enterica* serovar was converted to genome equivalents (GE) using a genome mass of 5.32 fg/GE based on *S. enterica* serovar Typhimurium ATCC 700720 [24,25]. Genome equivalents were calculated as previously described [26] using the following equation [26].

$$c_{\mathrm{target}}=\frac{n_{\mathrm{target}}\times c_{\mathrm{DNA}}\times N_{A}}{l_{\mathrm{DNA}}\times M_{\mathrm{bp}}}$$

Ten-fold serial dilutions ranging from 1.41 × 10^7^ to 1.41 GE/µL were prepared for individual serovars and mixed CKPI and TKPI panels. Each dilution was evaluated in triplicate under both singleplex and multiplex conditions. Standard curves were generated by plotting quantification cycle (Cq) values against log10 GE/µL, and linear regression analysis was used to determine slope, intercept, and coefficient of determination (R^2^). The linear dynamic range was defined as the concentration range exhibiting acceptable linearity (R^2^ ≥ 0.97) [27,28]. Amplification efficiency was calculated from the standard curve slope according to MIQE guidelines. Repeatability and intermediate precision were evaluated as described in Section 2.2.5. In addition, reaction-specific efficiency-corrected values were determined using LinRegPCR version 2021.2 [29,30,31].

#### 2.2.3. Limit of Quantification (LoQ)

The limit of quantification (LoQ) was defined as the lowest target concentration that could be consistently quantified with acceptable precision [27]. Five-fold serial dilutions were prepared from genomic DNA (30 ng/µL), corresponding to concentrations ranging from 5.73 × 10^6^ GE/µL to 0.587 GE/µL. qPCR was performed using three replicates twice daily over four consecutive days, resulting in 24 total replicates per target, including the genus-level *S. enterica* assay. The LoQ was defined as the lowest concentration yielding positive amplification in all replicates with acceptable quantitative precision, based on Cq coefficient of variation (CV) and repeatability criteria consistent with previously described precision studies [28,32].

#### 2.2.4. Limit of Detection (LOD)

The limit of detection (LoD95) was defined as the lowest target concentration estimated to produce positive amplification in 95% of reactions, as determined by probit regression analysis of detection outcomes obtained from 24 replicate reactions across serial dilutions [32]. LoD95 analysis was performed using the same dilution series and replicate structure described for LoQ determination.

#### 2.2.5. Precision (Repeatability and Intermediate Precision)

Precision was evaluated in terms of repeatability (intra-assay precision) and intermediate precision (inter-run precision) using replicate data generated during the LoD and LoQ studies. Repeatability was assessed under identical operating conditions, including the same analyst, instrument, laboratory, and experimental run. Intermediate precision was evaluated using independent experimental runs performed on different days within the same laboratory using the same instrument and operator. Inter-laboratory reproducibility was not evaluated in the present study. Precision was expressed as the standard deviation (SD) and coefficient of variation (CV) of replicate Cq values. Assay precision was considered acceptable when CV values remained within established qPCR validation criteria across the evaluated quantitative range [33,34].

#### 2.2.6. Inclusivity and Exclusivity (Analytical Specificity)

Analytical specificity of the GenoPATHX™ multiplex qPCR assay was evaluated using inclusivity and exclusivity testing across 128 bacterial isolates, including 98 *Salmonella enterica* isolates and 30 closely related non-*Salmonella* species. Inclusivity assessed correct detection of intended target serovars, whereas exclusivity evaluated absence of amplification among non-target organisms.

#### 2.2.7. Assay Robustness

Assay robustness was evaluated by introducing controlled variations in annealing temperature (63 °C, 64 °C, and 65 °C) and DNA template input volume (2 µL, 3 µL, and 4 µL). All reactions were performed in triplicate. The effects of experimental variation on assay performance were analyzed using linear mixed-effects models with experimental condition treated as a fixed effect and assay target and fluorophore treated as random effects. Tukey-adjusted estimated marginal means were used for pairwise comparisons.

#### 2.2.8. Evaluation of Assay Performance in Artificially Inoculated Matrices

The performance of the GenoPATHX™ multiplex qPCR assay was evaluated in artificially inoculated chicken rinsate (CKPI), ground turkey (TKPI), and ground beef matrices using direct DNA extraction without enrichment. Mixed *Salmonella enterica* serovar inocula were prepared using *S.* Typhimurium, *S.* Enteritidis, and monophasic *S*. Typhimurium for the CKPI panel, and *S.* Typhimurium, *S.* Muenchen, and *S.* Hadar for the TKPI panel. The genus-level *Salmonella enterica* target represented detection of the combined inoculated serovar. Overnight cultures prepared as described in Section 2.1 were adjusted to approximately 0.5 McFarland (≈1.5 × 10^8^ CFU/mL) and combined in equal volumes (1:1:1) to generate mixed-serovar inocula.

For the chicken rinsate and ground turkey experiments, 400 µL of the mixed-serovar inoculum was added to 1.6 mL of the respective matrix, resulting in a final volume of 2.0 mL and an initial bacterial concentration of approximately 3.0 × 10^7^ CFU/mL. Fresh chicken carcass rinsate was used directly as the liquid matrix, whereas the ground turkey matrix was prepared by combining fresh ground turkey with sterile buffered peptone water (BPW) to produce a homogeneous slurry. Following inoculation, samples were mixed thoroughly by vortexing and homogenized for approximately 1–2 min to ensure uniform distribution of the bacterial cells throughout the matrix. Five-fold serial dilutions were subsequently prepared in the corresponding uninoculated matrix (or sterile BPW for the homogenized meat slurry) to obtain bacterial concentrations ranging from approximately 3.0 × 10^7^ to 7.7 × 10^1^ CFU/mL. The initial inoculum concentration was selected to generate a broad serial dilution range spanning several orders of magnitude. This concentration range was used to comprehensively evaluate assay linearity, amplification efficiency, quantitative dynamic range, repeatability, intermediate precision, and analytical sensitivity. Lower bacterial concentrations generated through serial dilutions were included in the analytical validation and formed the basis for determination of the limit of quantification (LoQ) and the 95% limit of detection (LoD95).

For the ground beef experiments, 120 µL of each dilution was added directly to 6 g portions of ground beef, followed by the addition of sterile PBS to facilitate homogenization. Samples were thoroughly mixed to produce a uniform suspension before processing. Aliquots designated for molecular analysis were processed immediately following inoculation and homogenization to minimize changes in bacterial concentration during sample handling. Samples were subjected to sequential centrifugation at 500× *g* for 10 min, followed by 4430× *g* for 10 min, prior to DNA extraction using a modified PrepMan™ Ultra protocol incorporating Proteinase K treatment and heat lysis [35].

Extracted DNA was analyzed using the GenoPATHX™ (QuantiPATH Bio, Denver, CO, USA) multiplex qPCR assay on the Bio-Rad CFX Opus 96 Real-Time PCR System (Bio-Rad Laboratories, Inc., Hercules, CA, USA). Parallel aliquots were serially diluted and plated in triplicate on XLD agar to determine viable bacterial concentrations (CFU/mL or CFU/g), which served as the reference for quantitative performance evaluation. Selected dilutions were evaluated by qPCR, and all experiments were performed using three independent biological replicates. Uninoculated matrices previously confirmed *Salmonella*-negative were included as negative controls.

#### 2.2.9. Matrix Effect Evaluation (PBS vs. Chicken Rinsate)

Five-fold serial dilutions were prepared in both phosphate-buffered saline (PBS) and chicken rinsate matrices using standardized bacterial cultures (~10^8^ CFU/mL), as described in Section 2.2.8. Viable bacterial concentrations were confirmed by plate enumeration, and DNA extraction was performed in triplicate for each matrix.

Standard curves were generated by plotting Cq values against log10 CFU/mL, and amplification slope, intercept, efficiency, and coefficient of determination (R^2^) were compared between matrices. Generalized least squares (GLS) models were used to evaluate matrix-associated differences in amplification performance and Cq shift between PBS and chicken rinsate. This experiment was conducted to evaluate the effect of poultry-associated matrix components on qPCR amplification performance relative to PBS.

### 2.3. Short Enrichment Study

To evaluate the effect of short non-selective enrichment on assay sensitivity, serial dilutions corresponding to approximately 1.5 × 10^4^ to 1.5 × 10^3^ CFU/mL (10^−4^ and 10^−5^ dilutions) were incubated in Actero™ enrichment medium at 37 °C for 3 h and 6 h. Following enrichment, DNA was extracted using PrepMan™ Ultra reagent (Applied Biosystems, Foster City, CA, USA), and 3 µL of extracted DNA was used in 25 µL multiplex qPCR reactions. Amplification was performed on the Bio-Rad CFX Opus 96 Real-Time PCR System (Bio-Rad Laboratories, Hercules, CA, USA), and Cq values obtained after 3 h and 6 h enrichment were compared to evaluate the effect of short enrichment duration on assay sensitivity.

### 2.4. Field Evaluation of Diagnostic Performance

Field samples were collected from four poultry houses within a single commercial poultry farm and included litter (*n* = 16), feces (*n* = 16), boot swabs (*n* = 4), feed (*n* = 4), and water (*n* = 4). The four poultry houses represented separate sampling locations within a single production site rather than independent commercial farms. Litter and fecal samples were collected from four quadrants within each house, whereas one boot swab, one feed sample, and one water sample were collected from each poultry house.

Samples were evaluated using two analytical workflows: (i) direct molecular detection without enrichment for same-day quantification and (ii) a 3 h non-selective Actero™ enrichment at 37 °C with shaking to evaluate the effect of short enrichment on assay sensitivity. Results from both workflows were compared with the USDA-FSIS MLG 4.15 reference culture method [36].

Solid matrices, including feces, litter, feed (10 g), and boot swabs, were homogenized in phosphate-buffered saline (PBS) using a stomacher (230 rpm, 2 min). Water samples (30 mL) were processed directly. Homogenized samples were then aliquoted for direct molecular detection, short enrichment, and culture-based analysis.

For molecular workflows, 30 mL aliquots were subjected to sequential centrifugation at 500× *g* for 10 min followed by 4430× *g* for 10 min. Pellets were resuspended in PrepMan™ Ultra reagent (400 µL) supplemented with Proteinase K (20 µL), subjected to heat lysis at 100 °C for 20 min and clarified by centrifugation at 14,000 rpm. The clarified supernatant containing the extracted DNA was carefully transferred to a clean microcentrifuge tube. DNA concentration and purity were determined using a NanoDrop™ 2000c spectrophotometer (Thermo Fisher Scientific, Waltham, MA, USA) prior to qPCR analysis. Extracted DNA (3 µL) was used in 25 µL GenoPATHX™ multiplex TaqMan qPCR reactions targeting CKPI serovars and the genus-level *S. enterica* target. Reference culture analysis was performed according to USDA-FSIS MLG 4.15 procedures [36], including buffered peptone water (BPW) pre-enrichment, selective enrichment in tetrathionate (TT) and Rappaport–Vassiliadis (RV) broths, and selective plating on DMLIA and XLD agar. Presumptive colonies were purified on Sheep Blood Agar and confirmed by whole-genome sequencing (WGS). Fluorescence thresholds and positivity criteria were established during analytical validation and applied consistently during diagnostic performance evaluation.

### 2.5. qPCR Reaction Conditions

All qPCR reactions were performed on the Bio-Rad CFX Opus 96 Real-Time PCR System (Bio-Rad Laboratories, Hercules, CA, USA) and analyzed using CFX Maestro™ software version 2.3. Reactions were conducted in a final volume of 25 µL using PrimeTime™ One-Step 4× Broad-Range Master Mix (Integrated DNA Technologies, Coralville, IA, USA) together with proprietary GenoPATHX™ primer and probe sets (QuantiPATH LLC, Denver, CO, USA). The GenoPATHX™ primer and probe sequences are proprietary intellectual property of QuantiPATH LLC and are therefore not publicly disclosed. However, the reference genome accession numbers used for assay design and in silico specificity analysis are provided in Supplementary Table S1, and complete reaction conditions, assay chemistry, analytical validation procedures, and performance characteristics are described herein to facilitate independent evaluation of assay performance. Thermal cycling conditions consisted of an initial denaturation step at 95 °C for 3 min, followed by 40 amplification cycles of 95 °C for 10 s and 64 °C for 30 s, with fluorescence acquisition performed during the 64 °C annealing/extension step. Amplification signals with Cq values ≤ 38 were considered positive based on thresholds established during analytical validation of the assay. All reactions included no-template controls (NTCs) and positive amplification controls.

### 2.6. Statistical and Data Analysis

All statistical analyses were performed in R (v4.4.3; R Foundation for Statistical Computing, Vienna, Austria). Amplification efficiency was calculated from linear regression analysis of Cq values versus log10 target concentration. Assay robustness experiments were analyzed using linear mixed-effects models with Tukey-adjusted pairwise comparisons. Comparisons between singleplex and multiplex assay formats were evaluated using linear models incorporating interaction terms, with statistical significance defined at α = 0.05. Quantitative agreement between methods was evaluated using bias, percent recovery, and fold-difference analyses. Inclusivity and exclusivity were calculated as the proportion of correctly classified target and non-target results, respectively, with exact Clopper–Pearson 95% confidence intervals (CIs). Statistical interpretation considered both statistical significance and biological relevance, particularly when small differences in Cq values were unlikely to influence diagnostic interpretation. Data processing, statistical analysis, and visualization were performed using the tidyverse, boot, broom, lme4, emmeans, and ggplot2 packages. Raw qPCR datasets and analytical outputs are provided in Supplementary Table S1.

The analytical validation strategy was developed in accordance with the Minimum Information for Publication of Quantitative Real-Time PCR Experiments (MIQE) guidelines and was further informed by the principles outlined in ISO 20395:2019, where applicable [37].

## 3. Results

### 3.1. In Silico Specificity Analysis

In silico specificity analysis demonstrated high target specificity for all GenoPATHX™ primer sets. BLASTn analysis, with a maximum retrieval of 100 sequence hits per query, showed that the *S.* Enteritidis and *S.* Muenchen assays aligned exclusively with their intended target serovars. The *S.* Hadar assay exhibited limited sequence similarity to a single *S.* Anatum sequence, whereas the *S.* Typhimurium assay showed sequence similarity to *S.* Albert and monophasic *S.* Typhimurium. As expected, the monophasic *S.* Typhimurium assay also aligned with *S.* Typhimurium because of the close genetic relationship between these variants. No significant sequence similarity to non-*Salmonella* organisms was identified for the genus-level *S. enterica* assay.

### 3.2. Amplification Efficiency, Linearity and Linear Dynamic Range

**Figure 1:**
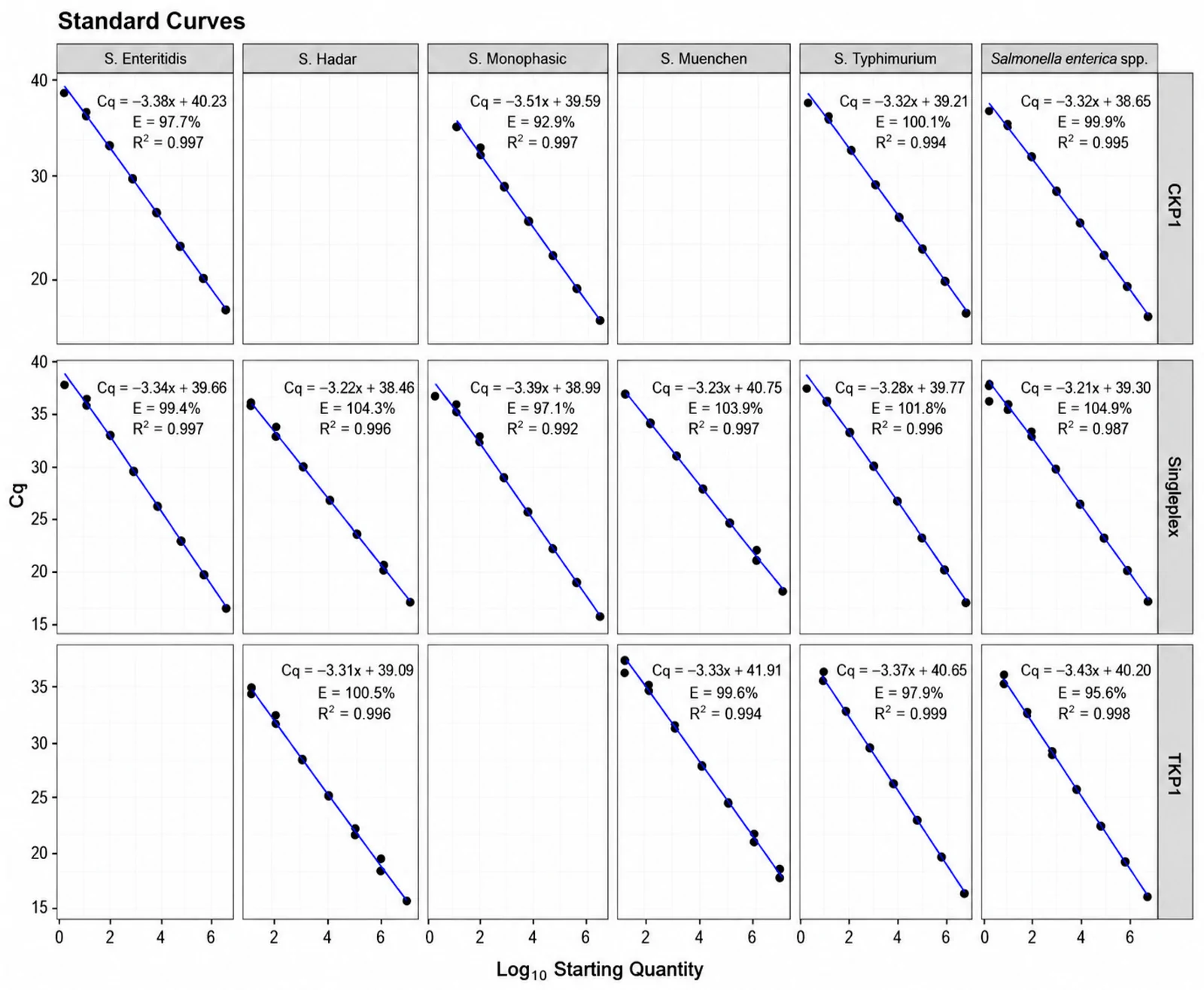
Standard curves (blue line) and amplification performance of the GenoPATHX™ multiplex qPCR assay for CKPI and TKPI targets (black circles). Standard curves were generated using serial dilutions of target DNA or bacterial suspensions. Representative standard curves illustrating amplification behavior and assay linearity for the CKPI and TKPI panels. Detailed numerical values for amplification efficiency, regression slope, intercept, and R^2^ are presented in Table 1.

**Figure 2:**
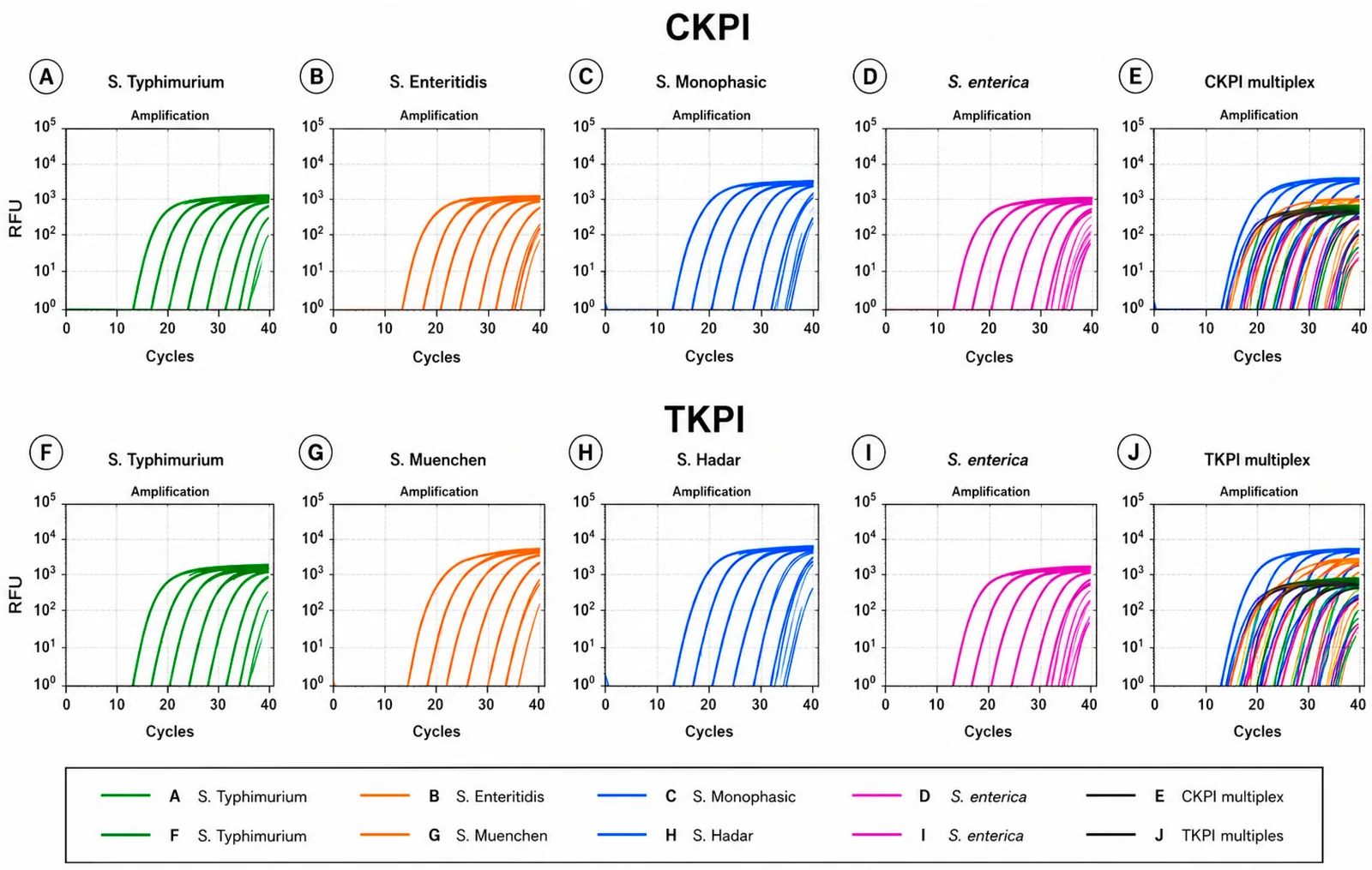
Amplification-efficiency analysis of the GenoPATHX™ multiplex qPCR assay using LinRegPCR. Reaction-specific amplification efficiencies were calculated from individual fluorescence amplification curves. The same standard dilution series of *S.* Typhimurium and *S. enterica* was used to evaluate both the CKPI and TKPI multiplexes. (**A**,**F**) *S.* Typhimurium; (**B**) *S.* Enteritidis; (**C**) monophasic *S.* Typhimurium; (**D**,**I**) *S. enterica*; (**E**) CKPI multiplex amplification; (**G**) *S.* Muenchen; (**H**) *S.* Hadar; and (**J**) TKPI multiplex amplification. Summary numerical values are provided in Table 2.

The GenoPATHX™ multiplex qPCR assay demonstrated consistent amplification performance in both singleplex and multiplex formats, indicating that simultaneous target detection did not significantly affect amplification efficiency or linearity. Across all targets and assay configurations, standard curves exhibited excellent linearity (R^2^ = 0.987–0.999) and amplification efficiencies within generally acceptable quantitative ranges (92.9–104.9%), with a broad linear dynamic range spanning approximately 10^1^ to 10^7^ GE/reaction (Table 1; Figure 1 and Figure 2).

Standard curve slopes ranged from −3.21 to −3.53 for singleplex assays, −3.32 to −3.51 for the CKPI multiplex panel, and −3.31 to −3.43 for the TKPI multiplex panel, indicating consistent amplification kinetics across assay formats. Replicate variability remained low throughout the evaluated concentration range, with standard deviation (SD) values generally below 0.4 Cq and mean coefficient of variation (CVs) ≤ 1.44%. No statistically significant differences in amplification slopes were observed between singleplex and multiplex formats (*p* ≥ 0.134), further supporting the analytical performance of the multiplex assay.

Efficiency-corrected quantification using LinRegPCR yielded mean PCR efficiencies ranging from 1.98 to 2.2 across all targets, with most assays approaching the theoretical amplification efficiency of 2.0 (Table 2). Correlation coefficients remained consistently high (R^2^ = 0.993–1.000), supporting reliable efficiency-corrected quantification across all assay targets.

Detailed amplification performance metrics are presented in Table 1 and Table 2 and Supplementary Table S1.

Efficiency-corrected analysis using LinRegPCR yielded mean PCR efficiencies ranging from 1.98 to 2.2 across all evaluated serovars, with most assays approaching the theoretical amplification efficiency of 2.0 (100% efficiency) (Table 2). Correlation coefficients remained consistently high (R^2^ = 0.993–1.000), supporting reliable amplification kinetics and consistent efficiency-corrected quantification across all assay targets.

### 3.3. Limit of Quantification (LoQ)

The GenoPATHX™ multiplex qPCR assay demonstrated target-dependent limits of quantification (LoQ) ranging from 43.8 to 219 GE/reaction (Table 3). *S.* Enteritidis and *S.* Typhimurium exhibited the lowest LoQ values (43.8 GE/reaction), whereas *S.* Hadar, monophasic *S.* Typhimurium, and *S.* Muenchen demonstrated LoQ values of 219 GE/reaction. All targets achieved 100% positive detection at their respective LoQ with acceptable quantitative precision, as indicated by coefficients of variation (CVs) ranging from 2.34% to 9.36%. At concentrations below the established LoQ, detection became progressively less consistent across all assay targets.

### 3.4. Limit of Detection (LoD95)

**Figure 3:**
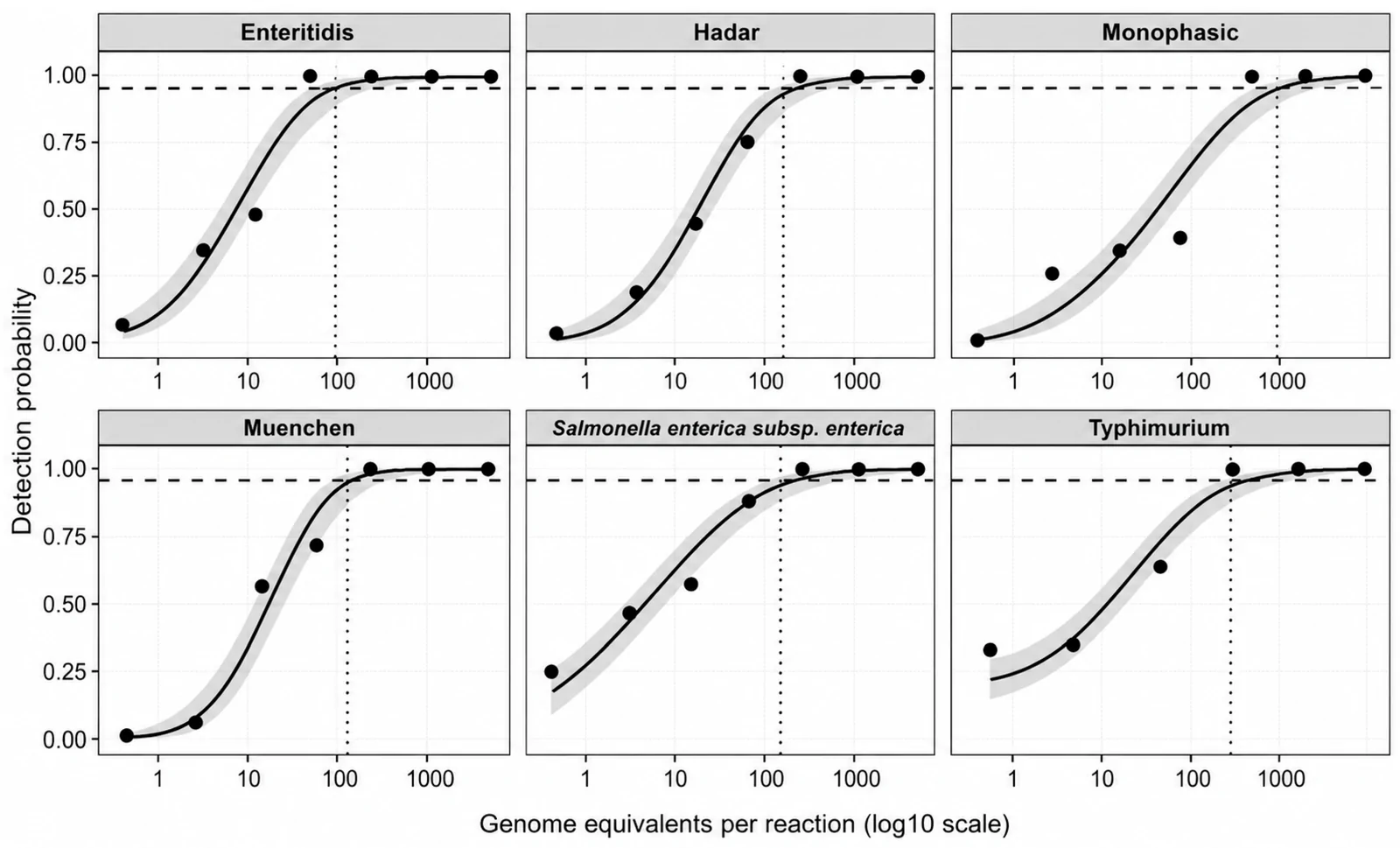
Limit of detection (LoD95) analysis of the GenoPATHX™ multiplex qPCR assay across evaluated *Salmonella* targets. Detection probability curves were generated using serially diluted target concentrations to estimate LoD95 values for each target. LoD95 represents the lowest concentration predicted to produce positive amplification in 95% of reactions. Error bars represent 95% confidence intervals.

The GenoPATHX™ multiplex qPCR assay demonstrated target-dependent limits of detection (LoD95) ranging from 60.0 to 545 GE/reaction (Table 4 and Figure 3). *S.* Typhimurium (60.0 GE/reaction) and *S.* Enteritidis (71.2 GE/reaction) exhibited the highest analytical sensitivity, followed by *S.* Muenchen (116 GE/reaction) and the genus-level *S. enterica* target (118 GE/reaction). *S.* Hadar demonstrated an LoD95 of 168 GE/reaction, whereas monophasic *S.* Typhimurium exhibited the highest LoD95 values (545 GE/reaction). Detection became progressively less consistent below the estimated LoD95 threshold values, consistent with the expected stochastic behavior of target detection near the analytical limit of detection. Detailed LoD95 results are provided in Supplementary Table S2 (LoD_LoQ_Precision).

### 3.5. Precision (Repeatability and Intermediate Precision)

The GenoPATHX™ multiplex qPCR assay demonstrated high quantitative precision across all evaluated targets and concentration ranges. At concentrations ≥ LoQ, intra-assay precision (repeatability) was high, with coefficients of variation (CVs) ranging from 1.07% to 9.52% (Table 5). The lowest repeatability-associated variability was observed for *S.* Typhimurium (~1.07%), *S.* Enteritidis (~1.95%), and the genus-level *S. enterica* target (~2.21%), whereas slightly higher, yet acceptable variability was observed for *S.* Hadar and monophasic *S.* Typhimurium.

Intermediate precision (inter-run precision) demonstrated comparable performance, with CVs at concentrations ≥ LoQ ranging from 1.19% to 9.36% across assay targets. Variability increased modestly at concentrations below the LoQ, consistent with the stochastic amplification effects expected near the lower limit of quantification in qPCR assays.

### 3.6. Inclusivity and Exclusivity (Analytical Specificity)

Analytical specificity of the GenoPATHX™ multiplex qPCR assay was evaluated using 128 bacterial isolates, including 98 *Salmonella enterica* isolates representing target and non-target serovars and 30 non-*Salmonella* species. The genus-level *Salmonella* assay demonstrated complete analytical inclusivity (98/98, 100.0%; 95% CI: 96.3–100.0) and complete analytical exclusivity (30/30, 100.0%; 95% CI: 88.4–100.0). These results demonstrate accurate detection of all *Salmonella* isolates and no amplification of the non-*Salmonella* species included in the analytical specificity panel (Table 6).

All evaluated target isolates were correctly identified by their corresponding serovar-specific assays, including *S.* Typhimurium (1/1), *S.* Enteritidis (1/1), *S.* Hadar (1/1), *S.* Muenchen (3/3), and monophasic *S.* Typhimurium 1,4,[5],12:i:- (3/3), with corresponding exact 95% confidence intervals reported in Table 6. Analytical exclusivity of the serovar-specific assays ranged from 93.7% to 97.6%, with no evidence of substantial amplification among evaluated non-target isolates.

Detailed analytical specificity results are provided in Supplementary Table S3 (Inclusivity and Exclusivity).

### 3.7. Assay Robustness

#### 3.7.1. Effect of Annealing Temperature

Assay robustness was evaluated by examining the effects of annealing temperature and DNA input volume on amplification performance. Summary statistics are presented in Table 7 and Table 8. Annealing temperatures of 63 °C, 64 °C, and 65 °C were evaluated across six assay targets and four fluorophore channels (*n* = 216 reactions). Mean Cq values remained stable across the evaluated temperatures, ranging from 30.58 ± 2.01 to 31.15 ± 2.07, with coefficients of variation (CVs) ranging from 6.40% to 6.65% (Table 7). Linear mixed-effects analysis identified a statistically significant overall effect of annealing temperature (F(2, 205) = 11.66, *p* < 0.0001). However, Tukey-adjusted pairwise comparisons showed no significant difference between 63 °C and 64 °C (*p* = 0.126).

Although amplification at 65 °C resulted in a modest increase in Cq values relative to 64 °C (ΔCq = +0.34; 95% CI: 0.07–0.61; *p* = 0.013), the maximum model-estimated difference across the evaluated temperature range remained less than one amplification cycle. Collectively, these findings indicate that the GenoPATHX™ multiplex qPCR assay was robust to small variations in annealing temperature, with 64° selected as the optimal operating temperature for subsequent experiments.

#### 3.7.2. Effect of DNA Input Volume

DNA input volumes of 2–4 µL were evaluated across CKPI and TKPI multiplex panels (*n* = 72 reactions). Mean Cq values decreased modestly with increasing DNA volume, ranging from 33.22 ± 0.89 at 2 µL to 32.56 ± 1.18 at 4 µL (Table 8). Linear mixed-effects analysis identified a statistically significant effect of DNA input volume on amplification performance (F(2, 65) = 3.96, *p* = 0.0237); however, the maximum model-estimated difference across the evaluated input volumes was only 0.66 amplification cycles. These findings indicate that modest variation in DNA input volume had minimal practical impact on overall assay performance, demonstrating the robustness of the assay to small differences in template input. Accordingly, a DNA input volume of 3 µL was retained for all subsequent experiments.

### 3.8. Evaluation of Assay Performance in Artificially Inoculated Matrices

The performance of the GenoPATHX™ multiplex qPCR assay was evaluated in artificially inoculated chicken rinsate, ground turkey, and ground beef matrices using direct DNA extraction without enrichment. Five-fold serial dilutions were prepared for each matrix, and viable bacterial concentrations were confirmed by plate enumeration.

In chicken rinsate, the CKPI panel demonstrated significant linear amplification across the evaluated concentration range (3.0 × 10^7^ to approximately 7.68 × 10^1^ CFU/mL), with all regression models remaining statistically significant (*p* < 0.001; Table 9). Coefficients of determination (R^2^) ranged from 0.957 to 0.995, whereas amplification efficiencies ranged from 68.7% to 89.5%.

The TKPI panel evaluated in ground turkey demonstrated significant linear amplification across the tested concentration range (*p* < 0.001; Table 9). Coefficients of determination (R^2^) values ranged from 0.844 to 0.960, with amplification efficiencies ranging from 90.6% to 128.0%. The elevated amplification efficiencies observed for selected targets are discussed further in the Discussion and are interpreted as reflecting matrix-associated variability and standard curve estimation rather than true PCR amplification efficiency. Residual variability was moderately higher in ground turkey than in chicken rinsate, indicating greater matrix-associated variability in amplification performance.

Two-way ANOVA demonstrated significant effects of assay target and sample matrix on Cq values (*p* < 0.001), whereas the interaction between assay target and bacterial concentration was not statistically significant (F = 1.64, *p* = 0.151). These findings indicate that amplification slopes were generally consistent across multiplex targets within each assay panel. Detection consistency declined progressively with decreasing bacterial concentration across all matrices and assay targets, with the lowest reproducibly detected concentrations summarized in Table 10.

In directly inoculated ground beef, detection frequency decreased progressively with decreasing bacterial concentration (Table 11). For the CKPI panel, all three replicates were positive through dilution −4 (3/3), after which detection decreased to 1/3 positive replicates at dilutions −6 and −7. Correspondingly, mean Cq values increased from 34.99 ± 0.65 to 37.39. Similarly, the TKPI panel maintained complete detection through dilution −5 (3/3 replicates), followed by reduced detection at lower concentrations, with 2/3 positive replicates at dilution −6 and no detectable amplification at dilution −7. These findings demonstrate concentration-dependent detection behavior consistent with plate enumeration results and the expected stochastic effects associated with amplification at bacterial concentrations approaching the assay’s lower detection limit.

### 3.9. Matrix Effect Evaluation (PBS vs. Chicken Rinsate)

**Figure 4:**
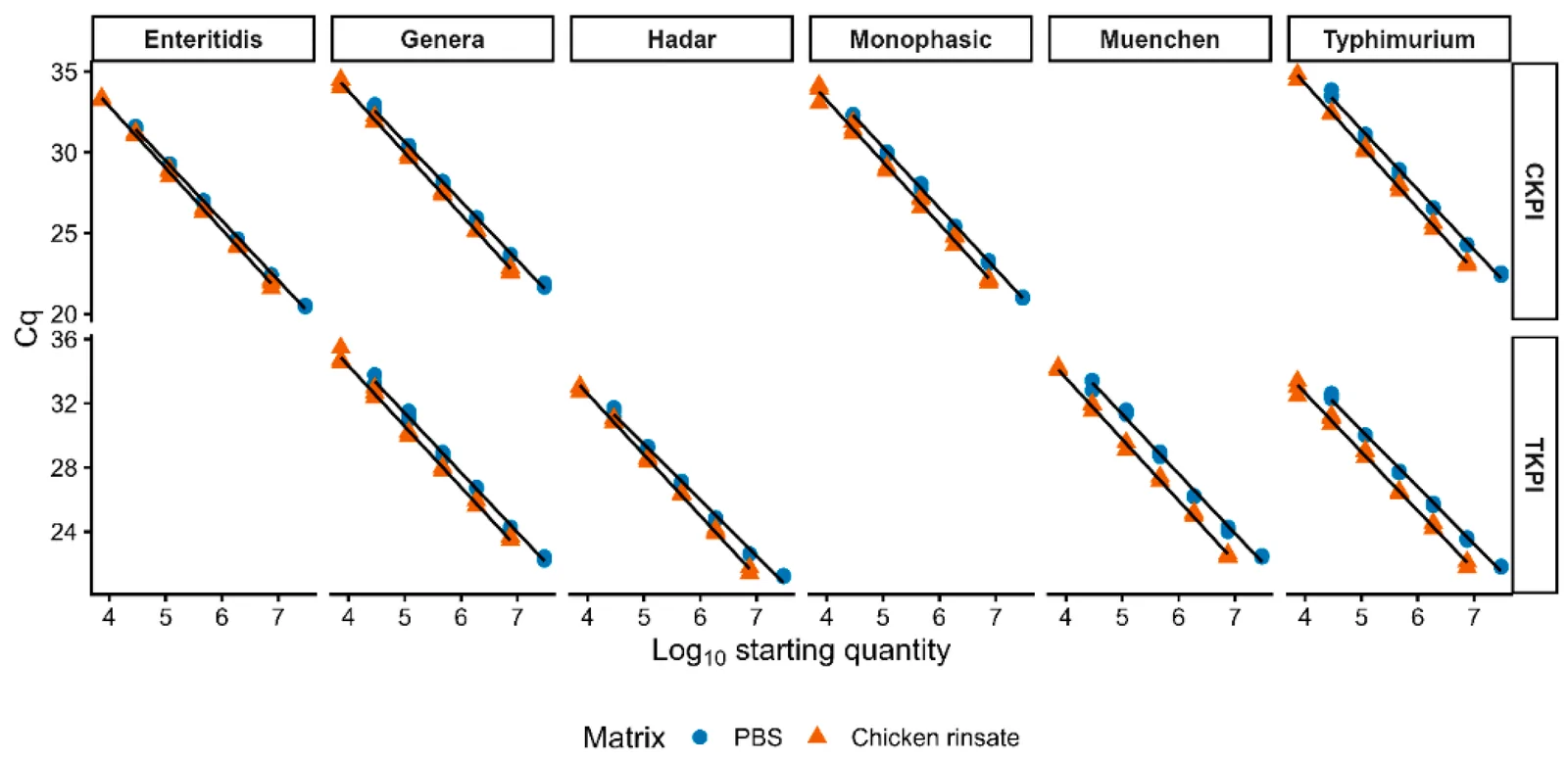
Matrix-effect evaluation of the GenoPATHX™ multiplex qPCR assay in phosphate-buffered saline (PBS) and chicken rinsate. Comparative standard curves are shown for the evaluated multiplex targets. Cq values were plotted against the log10 starting bacterial quantity. Blue circles represent PBS samples, orange triangles represent chicken-rinsate samples, and black lines represent fitted linear regression models. Although modest matrix-associated shifts in amplification were observed, the regression lines remained highly parallel between matrices, supporting the preservation of quantitative amplification behavior.

Standard curves generated in phosphate-buffered saline (PBS) and chicken rinsate demonstrated excellent linearity across all evaluated targets (R^2^ = 0.995–0.999), indicating robust quantitative amplification performance in both matrices (Figure 4). PCR amplification efficiencies ranged from 84.3% to 93.6% in PBS and from 81.4% to 87.2% in chicken rinsate (Table 12). Although amplification efficiencies were modestly lower in chicken rinsate than in PBS, all targets exhibited statistically significant linear amplification across the evaluated concentration range.

Generalized least squares (GLS) modeling was used to evaluate matrix-associated differences in amplification slope and Cq values between phosphate-buffered saline (PBS) and chicken rinsate. Statistically significant differences were identified for the CKPI targets *S.* Enteritidis, *S. enterica* spp., and *S.* Typhimurium, as well as the TKPI target *S.* Hadar (FDR-adjusted *p* ≤ 0.029; Table 12). No statistically significant slope differences were observed for the remaining targets (FDR-adjusted *p* ≥ 0.151).

Estimated matrix-associated ΔCq values (chicken rinsate − PBS) ranged from −0.51 to −1.64 cycles. For targets exhibiting significant matrix effects, the corresponding 95% confidence intervals did not include zero, indicating consistent differences in amplification behavior between the two matrices. Despite these differences, regression lines remained highly parallel across matrices (Figure 4), and PCR amplification efficiencies remained within acceptable ranges for quantitative analysis.

Overall, the observed ΔCq shifts (<2 cycles) indicate modest matrix-associated effects on amplification rather than substantial qPCR inhibition, supporting the suitability of the assay for quantitative analysis across the evaluated poultry-associated matrices. Detailed statistical results are provided in Supplementary Table S4 (Matrix effect evaluation).

### 3.10. Short Enrichment Study

**Figure 5:**
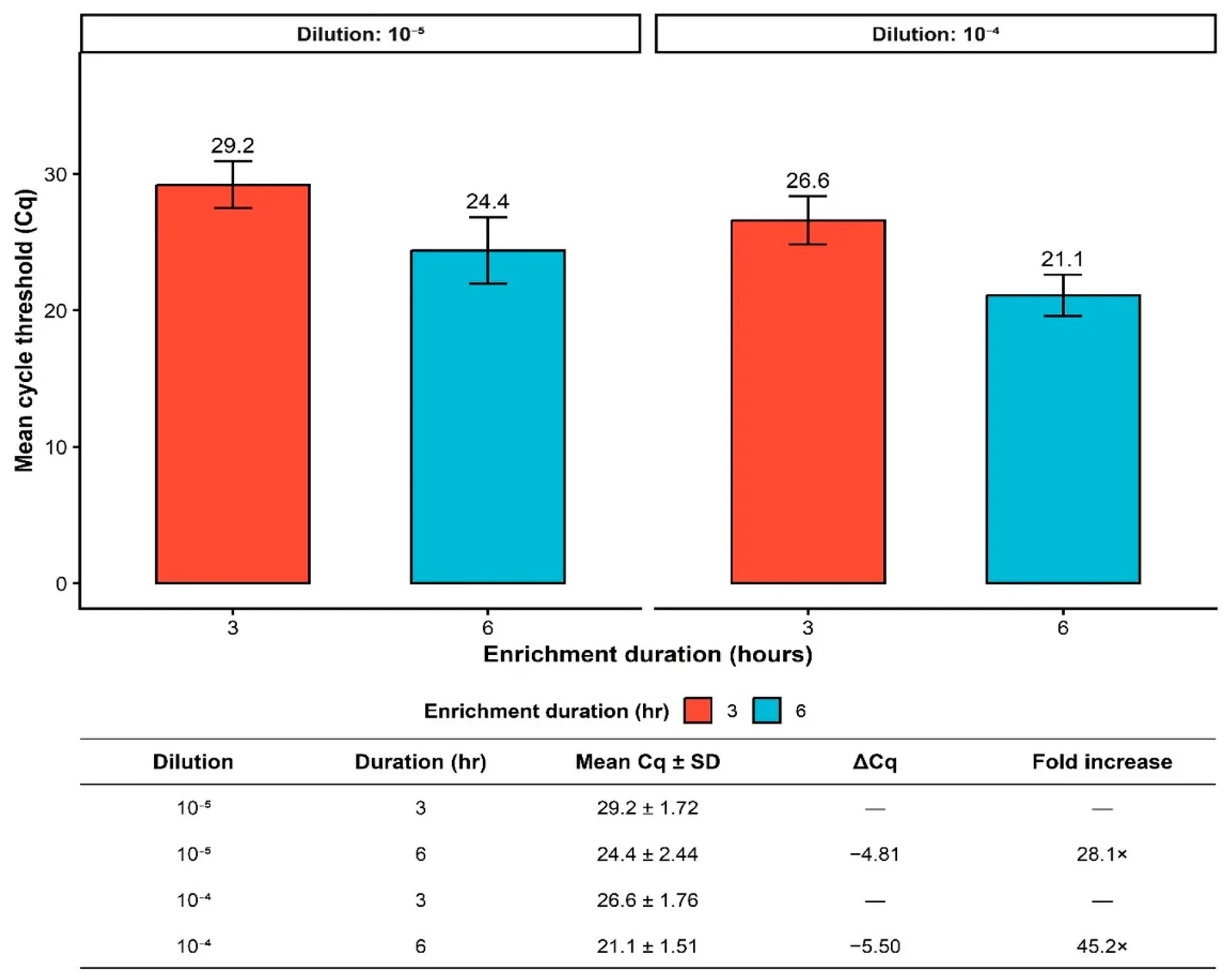
Effect of short enrichment duration on amplification performance of the GenoPATHX™ multiplex qPCR assay. Comparative amplification performance following 3 h and 6 h enrichment is shown across evaluated dilution levels. Mean Cq values decreased following extended enrichment, indicating increased detectable target signal and improved amplification sensitivity with longer enrichment duration.

Enrichment duration significantly affected amplification performance, with 6 h enrichment consistently producing lower Cq values than 3 h enrichment across the evaluated dilution levels (F = 456.49, *p* = 1.12 × 10^−9^; Figure 5). Dilution level also had a significant effect on amplification performance (F = 152.35, *p* = 2.24 × 10^−7^), and the overall linear model demonstrated excellent fit (R^2^ = 0.987).

At dilution 10^−5^, the mean Cq value decreased from 29.2 ± 1.72 following 3 h enrichment to 24.4 ± 2.44 following 6 h enrichment (ΔCq = −4.81). Similarly, at dilution 10^−4^, the mean Cq value decreased from 26.6 ± 1.76 to 21.1 ± 1.51 (ΔCq = −5.50). The corresponding increases in detectable target signal, estimated as 2^(–ΔCq), were approximately 28.1-fold and 45.2-fold for the 10^−5^ and 10^−4^ dilutions, respectively. All observed ΔCq values exceeded the technical variability established during assay precision studies.

Collectively, these findings demonstrate that short non-selective enrichment substantially enhanced detectable target signal while preserving the ability to achieve same-day molecular detection.

Detailed statistical outputs are provided in Supplementary Table S5 (Short enrichment study).

### 3.11. Preliminary Diagnostic Evaluation Using Field Samples

Field validation was conducted using 44 poultry environmental samples, including boot swabs (*n* = 4), feces (*n* = 16), litter (*n* = 16), water (*n* = 4), and feed (*n* = 4). Using the USDA-FSIS reference culture method, *Salmonella* was detected in 21 of 44 samples, corresponding to an overall prevalence of 47.7%. The GenoPATHX™ *S. enterica* spp. assay identified 19 of 44 samples (43.2%) as positive using the direct molecular workflow and 15 of 44 samples (34.1%) following 3 h Actero™ enrichment. Diagnostic performance relative to the reference culture method is summarized in Table 13, with corresponding confusion matrices presented in Table 14.

The direct molecular workflow demonstrated superior overall diagnostic performance compared with the 3 h Actero™ enrichment workflow, yielding a sensitivity of 81.0% (95% CI: 58.1–94.6%), specificity of 91.3% (95% CI: 72.0–98.9%), and overall accuracy of 86.4% (95% CI: 72.6–94.8%). Agreement with the reference culture method was substantial (κ = 0.73). In contrast, the 3 h Actero™ enrichment workflow demonstrated lower sensitivity (47.6%), specificity (78.3%), and overall accuracy (63.6%), with only fair agreement with the reference culture method (κ = 0.26).

Paired comparison of *S. enterica* spp. detection showed that the 3 h Actero™ enrichment workflow identified 15 positive samples, compared with 19 positive samples detected using the direct molecular workflow. Although the Actero™ workflow identified six additional detections among samples negative by direct extraction, it failed to detect ten samples identified as positive by the direct workflow. McNemar’s test demonstrated no statistically significant difference between workflows (*p* = 0.453).

**Figure 6:**
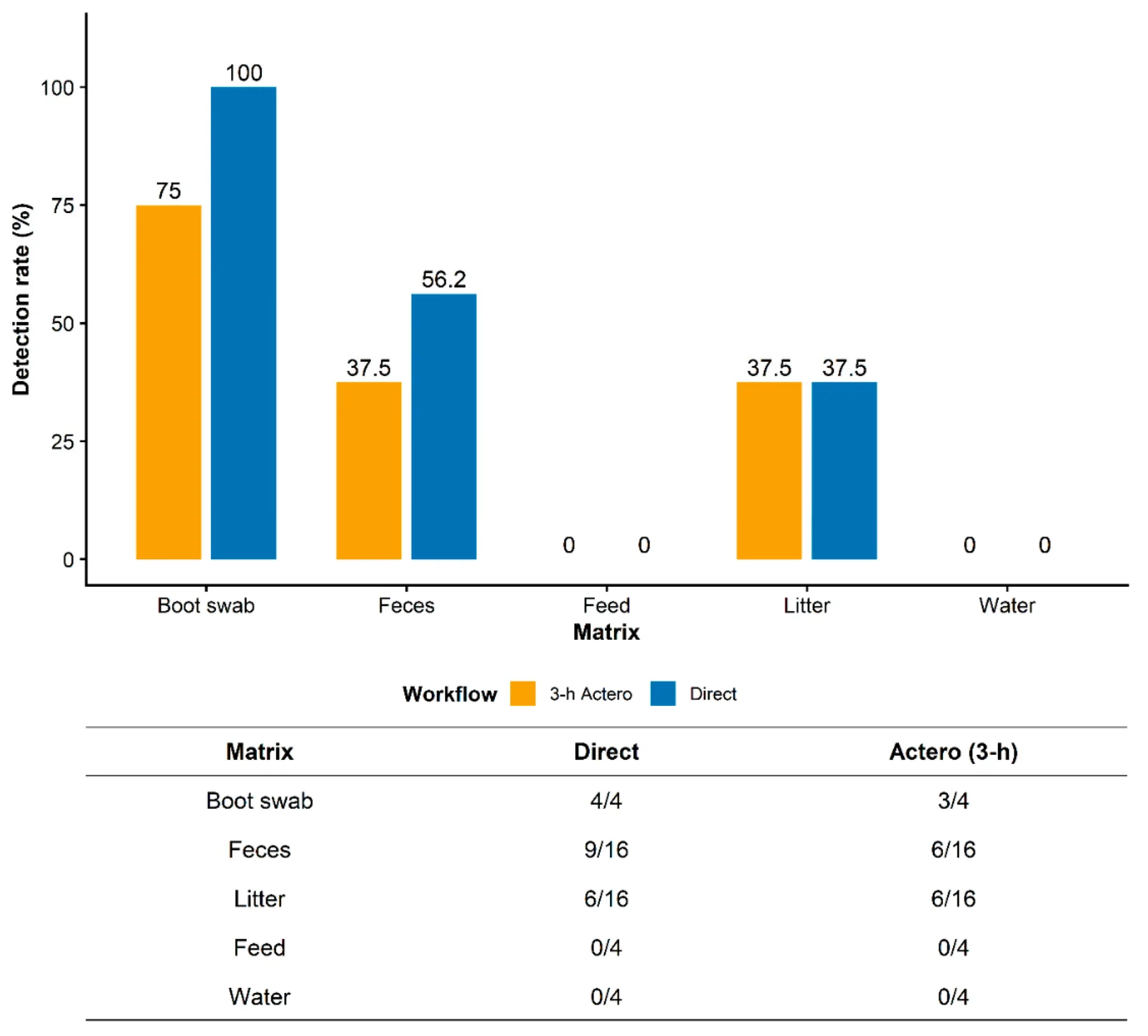
Matrix-specific detection frequencies of the GenoPATHX™ multiplex qPCR assay in poultry environmental samples. Detection frequencies obtained using the direct molecular workflow and the 3 h Actero™ enrichment workflow are shown across poultry environmental sample types, including boot swabs, feces, litter, water, and feed. Detection performance varied across matrices, with the largest reduction following short enrichment observed in fecal samples.

Matrix-specific detection frequencies are summarized in Figure 6. Boot swab samples were consistently positive using the direct molecular workflow (4/4), whereas the greatest reduction following Actero™ enrichment was observed in fecal samples, with detection decreasing from 9/16 (56.2%) to 6/16 (37.5%). Detection frequencies in litter samples were identical between workflows (6/16, 37.5%), whereas feed and water samples remained negative using both workflows.

Serovar-specific detections were limited across both workflows. The direct molecular workflow detected higher frequencies of *S.* Typhimurium (3/44), *S.* Enteritidis (2/44), and monophasic *S.* Typhimurium 1,4,[5],12:i:- (3/44) than the 3 h Actero™ enrichment workflow; however, none of these differences were statistically significant (*p* > 0.05).

Whole-genome sequencing (WGS) of recovered isolates identified *S.* Kentucky as the predominant environmental serovar (19/44; 43.2%), followed by *S.* Senftenberg (4/44; 9.1%) and *S.* Enteritidis (2/44; 4.6%). All samples identified as *S.* Enteritidis by WGS were also identified as *S.* Enteritidis using the GenoPATHX™ assay. Collectively, these findings demonstrate stronger overall field performance of the direct molecular workflow compared with the 3 h Actero™ enrichment workflow under the evaluated experimental conditions.

Detailed diagnostic validation results and WGS summaries are provided in Supplementary Table S6 (Diagnostic Validation) and Supplementary Table S7 (Whole Genome Sequencing).

## 4. Discussion

Rapid, accurate, and quantitative detection of *Salmonella* remains a priority for improving food safety surveillance and supporting evidence-based interventions throughout poultry production. Unlike conventional qPCR assays, which primarily provide qualitative positive-or-negative results, the GenoPATHX™ multiplex qPCR platform integrates serovar-specific detection, direct quantification, and same-day workflow capability within a single assay. In the present study, comprehensive analytical validation demonstrated that the assay achieved robust analytical sensitivity, quantitative performance, precision, analytical specificity, and operational robustness while maintaining favorable preliminary diagnostic performance in poultry environmental samples. This combined functionality is particularly relevant for modern poultry surveillance systems, where rapid identification and quantification of epidemiologically important serovars may support risk-based interventions, targeted processing decisions, and improved surveillance resolution.

### 4.1. In Silico Specificity Analysis

In silico specificity screening is an important preliminary step in molecular assay development because it enables rapid assessment of primer inclusivity and potential off-target sequence homology before experimental validation. In the present study, BLASTn analysis demonstrated high predicted specificity for all GenoPATHX™ primer sets. The *S.* Enteritidis and *S.* Muenchen assays aligned exclusively with their intended targets, whereas limited sequence homology was observed between the *S.* Hadar assay and a single *S*. Anatum sequence, and between the *S.* Typhimurium assay and *S.* Albert and monophasic *S.* Typhimurium. The monophasic *S.* Typhimurium assay likewise aligned with *S.* Typhimurium sequences, while the genus-level *S*. *enterica* assay demonstrated broad predicted inclusivity without detectable alignment to non-*Salmonella* organisms.

The observed sequence similarity between the *S.* Typhimurium and monophasic *S.* Typhimurium assays is biologically plausible given the close phylogenetic relationship and extensive genomic similarity between monophasic variants and classical *S.* Typhimurium lineages [38]. Importantly, sequence similarity identified by BLAST analysis does not necessarily predict amplification under experimental conditions, because primer performance is influenced by factors including mismatch position, thermodynamic stability, and PCR reaction kinetics [23,39,40]. Consequently, in silico analysis should be regarded as an initial screening approach and interpreted alongside experimental inclusivity and exclusivity data [41,42]. Consistent with the BLAST v2.17.0+ predictions, experimental testing demonstrated complete analytical inclusivity for the evaluated *Salmonella* isolate panel and complete analytical exclusivity against the evaluated non-*Salmonella* organisms, supporting the specificity of the GenoPATHX™ multiplex assay. The inclusion of both genus-level and serovar-specific targets further strengthens confidence in accurate target identification.

### 4.2. Amplification Efficiency, Linearity, and Dynamic Range

The GenoPATHX™ multiplex qPCR assay demonstrated excellent amplification linearity and quantitative performance across all assay panels, indicating that multiplexing did not substantially compromise amplification efficiency or analytical performance. Most PCR efficiencies remained within the MIQE-recommended range for quantitative PCR assays [43], whereas regression slopes and correlation coefficients were highly consistent between singleplex and multiplex formats, supporting stable amplification kinetics and reliable target quantification [44,45,46]. Similar performance has been reported for other multiplex qPCR assays developed for the simultaneous detection of foodborne pathogens and serovar-specific targets [47,48].

The analytical validation strategy adopted in this study was guided by the MIQE recommendations and, where applicable, by the principles outlined in ISO 20395:2019 for evaluating quantitative nucleic acid measurement methods.

No meaningful differences in amplification slopes were observed between singleplex and multiplex reactions, suggesting minimal primer competition and limited fluorescence-associated interference, both of which are common analytical challenges in multiplex qPCR assays [49]. Furthermore, the low replicate variability observed across reactions (SD < 0.4; CV ≤ 1.44%) demonstrated excellent repeatability and intermediate precision under optimized amplification conditions.

The broad linear dynamic range achieved across all assay targets supports the suitability of GenoPATHX™ for quantitative applications spanning multiple orders of magnitude. Efficiency-corrected quantification showed close agreement between predicted and expected genome equivalents, indicating minimal quantification bias and consistent amplification performance across multiplex targets. These characteristics are particularly important for multiplex diagnostic assays, in which differences in target amplification efficiency can influence quantitative interpretation.

Reaction-specific efficiency analysis using LinRegPCR further confirmed the stability of amplification kinetics across all evaluated targets. Mean PCR efficiencies ranged from 1.98 to 2.20, with most reactions approaching the theoretical optimum of 2.0, corresponding to complete target doubling during each amplification cycle [29]. High reaction-level correlation coefficients (R^2^ = 0.993–1.000) further supported the reliability of efficiency-corrected quantification [50]. Unlike conventional standard curve-derived estimates, LinRegPCR evaluates amplification efficiency at the individual reaction level and can provide additional insight into reaction-specific variability and subtle amplification inhibition. The close agreement between conventional standard curve analysis and LinRegPCR-derived efficiencies therefore provides independent confirmation of the quantitative reliability and analytical robustness of the GenoPATHX™ assay.

Although amplification efficiencies for selected targets in the ground turkey matrix exceeded the commonly accepted qPCR range (90–110%), these estimates were derived from matrix-based standard curves generated using artificially inoculated food samples rather than assay optimization experiments. Standard curve-derived efficiencies are influenced by regression characteristics and may be affected by complex food matrices, limited dilution series, and biological variability, occasionally producing apparent efficiencies greater than 100%. Importantly, LinRegPCR analysis yielded reaction-specific efficiencies that remained close to the theoretical optimum for all assay targets, indicating that the elevated standard curve-derived efficiencies most likely reflected regression variability rather than aberrant PCR amplification. The consistently high linearity, reproducible amplification, and favorable diagnostic performance observed throughout the study further support the analytical robustness of the GenoPATHX™ multiplex qPCR assay.

### 4.3. LoQ and LoD of the Assay

The GenoPATHX™ multiplex qPCR assay demonstrated high analytical sensitivity and robust quantitative performance, with target-specific limits of quantification (LoQ) ranging from 43.8 to 219 genome equivalents (GE)/reaction and LoD95 values ranging from 60.0 to 545 GE/reaction. All evaluated targets achieved 100% detection at their respective LoQ concentrations while maintaining acceptable quantitative precision (CV: 2.34–9.36%), supporting reliable quantitative amplification across the multiplex system. These coefficients of variation are consistent with values generally considered acceptable for qPCR-based quantification, particularly near the lower quantification where variability is expected to increase because of stochastic amplification at low target concentrations [33,51]. Comparable low-level detection performance has also been reported for other molecular amplification platforms, including loop-mediated isothermal amplification (LAMP), where limits of detection of approximately 75 cells/reaction have been described [52].

Among the evaluated targets, *S.* Typhimurium and *S.* Enteritidis exhibited the lowest LoQ and LoD95 values, indicating greater analytical sensitivity than the remaining assay targets. In contrast, the monophasic *S.* Typhimurium assay exhibited the highest LoD95 and among the highest LoQ values within the multiplex panel. This difference may, in part, reflect target-specific assay characteristics, including the relatively larger amplicon size of the monophasic assay compared with the other evaluated targets [53]. Longer qPCR amplicons have been associated with reduced amplification efficiency and analytical sensitivity, particularly at low template concentrations and under multiplex conditions where multiple targets compete for shared reaction components [54].

The LoD95 values observed in the present study are comparable to those reported for other molecular assays developed for *Salmonella* detection using purified DNA or low cell-equivalent standards. GenoPATHX™ detected targets at 60.0–545 GE/reaction, with the greatest analytical sensitivity observed for *S.* Typhimurium and *S.* Enteritidis, which were detected at 60.0 and 71.2 GE/reaction, respectively. Previous studies have reported qPCR detection limits ranging from approximately 10^0^–10^2^ CFU/reaction for multiplex assay targeting poultry-associated *Salmonella* serovars and carcass contamination [23,55,56], while other TaqMan^®^ real-time PCR assays have achieved detection limits of approximately 10^2^ CFU/mL in pure culture systems [47]. Although direct comparison should be interpreted cautiously because of differences in DNA extraction methods, amplification chemistries, target selection, sample matrices, and reporting units, the analytical sensitivity observed for GenoPATHX™ falls within the low-copy detection range reported for optimized *Salmonella* molecular assays.

The observed differences between LoQ and LoD95 across assay targets were expected because LoD95 represents the lowest concentration detected with a predefined probability threshold, whereas LoQ additionally requires acceptable quantitative accuracy, repeatability, and intermediate precision. At concentrations below the established LoQ thresholds, amplification became progressively more valuable across replicates, consistent with the stochastic amplification effects commonly observed in low-copy-number qPCR reactions.

Although regulatory testing for *Salmonella* is generally based on presence/absence criteria, analytical validation of a quantitative qPCR assay requires evaluation across a broad concentration range to establish key performance characteristics, including linearity, amplification efficiency, quantitative dynamic range, LoD_95_, and LoQ. Accordingly, relatively high starting inoculum concentrations were used solely to generate serial dilution series spanning both high and low bacterial loads. The lower concentrations within these dilution series were subsequently used to determine the analytical sensitivity of the assay, including the LoD_95_ and LoQ, thereby providing performance metrics relevant to low-level contamination.

Collectively, these findings demonstrate effective multiplex optimization and balanced reaction chemistry, enabling sensitive, reproducible, and quantitative detection of epidemiologically important *Salmonella* serovars relevant to poultry surveillance and food safety applications.

### 4.4. Inclusivity and Exclusivity (Analytical Specificity)

The GenoPATHX™ multiplex qPCR assay demonstrated strong analytical specificity, with the genus-level *Salmonella* assay achieving 100% analytical inclusivity and 100% analytical exclusivity across the evaluated isolate panel. These results indicate accurate detection of all *Salmonella enterica* isolates included in the study, with no detectable amplification of the non-*Salmonella* organisms tested, supporting the suitability of the assay for highly specific molecular detection. High analytical inclusivity and exclusivity are essential performance characteristics of multiplex diagnostic assays because false-negative results may compromise pathogen surveillance, whereas non-specific amplification can reduce diagnostic accuracy, confidence, and assay reliability [57].

All serovar-specific assays likewise achieved complete analytical inclusivity for their intended targets, including *S.* Typhimurium, *S.* Enteritidis, *S.* Hadar, *S.* Muenchen, and monophasic *S.* Typhimurium 1,4,[5],12:i:-. These findings indicate that the selected primer–probe sets successfully targeted conserved yet discriminatory genomic regions capable of differentiating epidemiologically important *Salmonella* serovars within a multiplex format. Similar multiplex qPCR studies have reported high analytical specificity when carefully selected serovar-associated genetic targets are employed [58]. Likewise, the strong genus-level specificity observed in the present study is also consistent with previous reports demonstrating that optimized probe-based qPCR assays can achieve excellent discrimination between *Salmonella* and closely related members of the *Enterobacteriaceae* family [47,59].

Although complete analytical inclusivity and exclusivity were achieved for the isolate panel evaluated in this study, the tested collection represents only a subset of the genetic diversity of *Salmonella* and non-*Salmonella* bacteria. Accordingly, these results should be interpreted as demonstrating excellent analytical specificity within the evaluated isolate panel rather than universal across the full diversity of circulating strains. Future evaluation using a larger, geographically and genetically diverse isolate collection would further strengthen confidence in the analytical specificity and broader application of the assay.

Although analytical exclusivity for the serovar-specific assays ranged from 93.7% to 97.6%, no substantial amplification of the evaluated non-target organisms was observed. The modest reductions in exclusivity likely reflect limited cross-reactivity among closely related *Salmonella* serovars that share homologous genomic regions, particularly *S.* Typhimurium and its monophasic variant, rather than amplification of unrelated bacterial species. Such target-dependent limitations are common in multiplex *Salmonella* assays because many serovars possess highly conserved genomic regions, making complete serovar-level discrimination inherently challenging within closely related groups [23]. Nevertheless, the absence of substantial non-specific amplification indicates that multiplexing did not meaningfully compromise the overall analytical specificity of the assay.

Collectively, these results demonstrate that the GenoPATHX™ multiplex qPCR assay provides highly specific and reliable detection of major poultry-associated *Salmonella* serovars while maintaining stable multiplex amplification performance.

### 4.5. Assay Robustness

Robustness testing demonstrated that the GenoPATHX™ multiplex qPCR assay maintained stable amplification across moderate variations in key reaction parameters, including annealing temperature and DNA input volume. Changes in annealing temperature (63–65 °C) and DNA input volume (2–4 µL) resulted in small but statistically significant shifts in Cq values (<1 amplification cycle), indicating that moderate deviations from the optimized protocol are unlikely to substantially affect assay interpretation or quantitative performance.

Despite these statistically detectable differences, amplification variability remained low across all evaluated targets and multiplex panels, demonstrating consistent assay performance under routine operating conditions. The limited magnitude of observed Cq shifts further supports the operational robustness of the assay and is consistent with expected behavior of well-optimized multiplex real-time PCR systems [43].

### 4.6. Evaluation of Assay Performance in Artificially Inoculated Matrices

The GenoPATHX™ multiplex qPCR assay demonstrated clear concentration-dependent amplification across all evaluated matrices, supporting the feasibility of direct quantitative detection without enrichment. In chicken rinsate, the assay exhibited strong quantitative performance, characterized by high linearity (R^2^ = 0.957–0.995) and low residual variability, indicating stable amplification and reliable quantification across the evaluated concentration range. These results are consistent with previous studies demonstrating accurate molecular quantification of *Salmonella* in poultry-associated liquid matrices [46].

In contrast, amplification performance in ground turkey exhibited greater variability and lower regression performance, likely reflecting the heterogeneous composition and increased inhibitory burden associated with comminuted meat matrices. Similar matrix-associated effects have been reported previously, with reduced amplification performance observed in the presence of high background microbial flora and complex food matrices [47]. Such reductions in linearity and increased variability are commonly attributed to PCR inhibitors, uneven bacterial distribution, and reduced DNA extraction efficiency.

Target-level analysis identified no statistically significant interaction between target identity and bacterial concentration, indicating comparable amplification slopes across serovars within each multiplex panel. These results demonstrate balanced multiplex amplification without evidence of substantial target-dependent quantitative bias. However, matrix-effect analysis revealed measurable differences in amplification kinetics between chicken rinsate and ground turkey, emphasizing the importance of matrix-specific calibration for accurate quantitative interpretation in complex food systems [60].

The observed LoQ values further reflected matrix-dependent assay performance. In chicken rinsate, LoQ values ranged from 11.4 to 56.6 CFU/mL, consistent with previously reported quantitative detection ranges for poultry-associated molecular assays [46]. Higher LoQ values in ground turkey, particularly for the genus-level assay, likely resulted from increased amplification variability and reduced efficiency associated with solid food matrices. Similar reductions in analytical sensitivity have been reported for multiplex *Salmonella* qPCR assays applied directly to unenriched meat samples compared with liquid or enrichment-based systems [47,55,61]. Accordingly, short enrichment strategies have been shown to improve low-level *Salmonella* detection by increasing recoverable bacterial numbers prior to amplification [47,55].

The GenoPATHX™ assay also demonstrated strong quantitative agreement with culture-based enumeration in chicken rinsate, with minimal systematic bias and consistent recovery across the evaluated dilution series. In ground beef, amplification remained concentration-dependent and generally consistent with culture enumeration, although greater variability was observed near the lower analytical detection range. This increased variability is expected at low bacterial concentrations and likely reflects stochastic sampling effects, heterogeneous bacterial distribution, limited template availability, and matrix-associated inhibition caused by fats, proteins, and other endogenous food components. Nevertheless, the assay maintained detectable amplification near its analytical detection limit, supporting the robustness of the GenoPATHX™ multiplex qPCR assay across diverse poultry-associated and complex food matrices.

### 4.7. Matrix Effect Evaluation (PBS vs. Chicken Rinsate)

The GenoPATHX™ multiplex qPCR assay maintained excellent amplification linearity in both phosphate-buffered saline (PBS) and chicken rinsate matrices (R^2^ = 0.995–0.999), demonstrating robust quantitative performance across the evaluated sample conditions. Although statistically significant differences in regression slopes and Cq values were observed for selected targets, the magnitude of these shifts remained small (<2 amplification cycles), indicating only minor matrix-associated effects rather than substantial PCR inhibition.

These observations are consistent with previous studies showing that poultry-associated matrices may introduce measurable but analytically minor variability without substantially compromising overall qPCR performance. The observed ΔCq shifts likely reflect differences in DNA recovery efficiency, matrix-associated fluorescence characteristics, and the presence of low levels of PCR inhibitors in chicken rinsate. Despite these effects, the regression lines remained highly parallel between matrices, indicating preservation of quantitative relationships across the evaluated concentration range.

Overall, these results demonstrate that the GenoPATHX™ multiplex qPCR assay maintains reliable quantitative performance in poultry-associated matrices while exhibiting only minimal matrix-related effects, supporting its suitability for direct quantitative analysis of poultry environmental samples.

### 4.8. Diagnostic Performance

The GenoPATHX™ multiplex qPCR assay demonstrated favorable preliminary diagnostic performance for the direct detection of *Salmonella* in poultry environmental samples. The direct molecular workflow outperformed the shortened 3 h Actero™ enrichment workflow in sensitivity, specificity, overall accuracy, and agreement with the reference culture method. Although enrichment is traditionally used to improve analytical sensitivity [62,63,64], shortening the enrichment period introduces a trade-off between turnaround time and bacterial recovery. The reduced performance observed following the 3 h enrichment workflow likely reflects insufficient recovery of low-level or sublethally injured *Salmonella* cells, together with increased interference from complex environmental matrices containing inhibitory compounds, organic debris, and competing microbiota [65].

The reduced diagnostic performance following the 3 h Actero™ enrichment is also consistent with the biology of *Salmonella* recovery during the early stages of enrichment. At low initial contamination levels, stressed or injured cells require time to recover before entering exponential growth. Furthermore, differences in recovery kinetics among serovars, combined with competition from background microbiota, may further limit bacterial proliferation during short enrichment periods. Consequently, bacterial populations may remain below the analytical detection threshold after only 3 h of incubation. Extending the enrichment period allows additional bacterial multiplication, resulting in higher target DNA concentrations, lower Cq values, and improved analytical sensitivity.

Although these results demonstrate the feasibility of direct molecular detection using the GenoPATHX™ platform, the field evaluation was conducted using environmental samples collected from four poultry houses within a single commercial poultry farm. Therefore, the reported diagnostic performance should be considered preliminary. Additional validation across multiple commercial farms, geographic regions, poultry production systems, and environmental conditions will be important to establish the broader applicability and diagnostic performance of the assay.

The present findings suggest that a 3 h enrichment period may be insufficient for optimal recovery of *Salmonella* from poultry environmental samples and that longer enrichment durations may be required to improve amplification consistency and analytical sensitivity [66]. Similar observations have been reported in previous studies, where extending enrichment substantially enhanced molecular detection in poultry-associated matrices, particularly under conditions of low-level contamination or environmentally stressed *Salmonella* populations [47,55,66]. The improved performance associated with longer enrichment has been attributed to increased bacterial recovery, reduced stochastic effects at low target concentrations, and greater template availability for downstream amplification.

The direct molecular workflow correctly identified 17 of 21 culture-positive samples while generating only two false-positive results, demonstrating substantial agreement with the USDA-FSIS reference culture method and supporting its potential for rapid, same-day surveillance applications. The limited number of false-negative results likely reflects low bacterial loads, heterogeneous distribution of *Salmonella* within environmental samples, or target concentrations approaching the assay’s lower limit of detection [55]. Together, these findings indicate that direct molecular testing can provide reliable diagnostic performance while substantially reducing the turnaround time associated with conventional culture-based methods.

Matrix-dependent differences in diagnostic performance were also observed. The greatest reduction in detection following short enrichment occurred in fecal samples, whereas litter samples demonstrated comparable performance between workflows. These observations are consistent with previous reports identifying fecal matrices as particularly challenging because of their high microbial burden, the presence of endogenous PCR inhibitors, and complex organic composition [5]. In contrast, boot swab samples consistently yielded high detection rates, likely because they provide a more representative measure of cumulative environmental contamination within poultry houses. Previous studies have similarly identified boot swabs (boot socks) as among the most reliable and reproducible environmental sampling methods for *Salmonella* surveillance in poultry production systems [67,68]. Collectively, these observations underscore the importance of matrix-specific optimization when implementing rapid molecular workflows for environmental surveillance.

Serovar-specific detection was limited across both workflows, likely reflecting low target abundance, mixed *Salmonella* populations, and the predominance of non-target serovars within the evaluated samples. Importantly, complete concordance between qPCR and whole-genome sequencing (WGS) for *S. Enteritidis*-positive samples supports the analytical specificity and serovar-level accuracy of the GenoPATHX™ assay targets. WGS analysis further identified *S.* Kentucky as the predominant environmental serovar, explaining the relatively low frequency of CKPI-target detections. This finding is consistent with previous reports identifying *S.* Kentucky as one of the most prevalent non-typhoidal *Salmonella* serovars in U.S. poultry production systems [69].

The dominance of *S.* Kentucky also highlights the importance of continuously adapting molecular surveillance panels to reflect regionally prevalent and epidemiologically emerging serovars [70]. Of particular concern, *S.* Kentucky sequence type 198 (ST198) has emerged globally as a multidrug-resistant lineage associated with high-level fluoroquinolone resistance and increasing public health significance [71,72,73,74]. These findings emphasize the value of flexible multiplex surveillance platforms that can be updated to incorporate regionally important serovars as the epidemiology of poultry-associated *Salmonella* evolves.

The favorable analytical and preliminary diagnostic performance of the GenoPATHX™ multiplex qPCR assay relative to the USDA-FSIS reference culture method supports its potential application as a rapid molecular tool for poultry surveillance and food safety monitoring. The USDA-FSIS reference culture method was selected as the diagnostic reference standard because it remains the regulatory benchmark for *Salmonella* detection in poultry. Although direct comparisons with commercially available molecular detection kits were beyond the scope of the present study, such evaluations would provide valuable information regarding comparative analytical performance, workflow efficiency, turnaround time, cost-effectiveness, and operational suitability for routine food safety testing. In addition, larger multi-site validation studies involving diverse poultry production systems and naturally contaminated samples will be important for confirming the robustness, generalizability, and practical utility of the GenoPATHX™ multiplex qPCR assay under routine surveillance conditions.

## 5. Overall Implications

Collectively, the findings of the present study demonstrate that the GenoPATHX™ multiplex qPCR assay provides a sensitive, quantitative, and adaptable platform for the detection of epidemiologically important *Salmonella* serovars across diverse poultry-associated matrices. The assay maintained robust analytical performance under multiplex conditions while supporting direct molecular detection and quantification without requiring extended enrichment.

The ability to generate quantitative, same-day molecular data offers a potential advantage over conventional presence/absence testing by supporting timelier, evidence-based decision-making for poultry production, environmental surveillance, and food safety monitoring. In addition to genus-level detection, the incorporation of serovar-specific targets enables simultaneous molecular characterization within a single multiplex workflow, thereby enhancing surveillance resolution and facilitating risk-based intervention strategies.

Although matrix-associated amplification variability and the influence of enrichment duration remain important considerations for practical implementation, the overall analytical and preliminary diagnostic performance demonstrated in this study supports the potential application of the GenoPATHX™ multiplex qPCR assay as a rapid molecular surveillance tool for poultry-associated *Salmonella* monitoring. Continued validation across broader sample types, poultry production systems, and independent laboratories will further establish its utility for routine food safety surveillance and regulatory applications.

## 6. Limitations

Several limitations should be considered when interpreting the findings of the present study. First, although all evaluated target isolates were correctly detected, the analytical inclusivity assessment included a limited number of isolates for certain serovars. Consequently, additional validation using geographically and genetically diverse isolate collections would further strengthen confidence in assay inclusivity and serovar-level specificity.

Second, the preliminary diagnostic evaluation was based on 44 environmental samples collected from a single commercial poultry farm, which may limit the generalizability of the findings to other poultry production systems, geographic regions, and environmental conditions. Accordingly, the diagnostic performance estimates reported here should be considered preliminary rather than a comprehensive validation according to ISO 16140 [37]. Future multi-site studies involving larger and more diverse sample sets are needed to further evaluate the assay performance under a broader range of field conditions.

Finally, although the GenoPATHX™ multiplex qPCR assay demonstrated robust analytical performance and encouraging preliminary diagnostic performance under the conditions evaluated, inter-laboratory validation was beyond the scope of the present study. Future collaborative studies involving independent laboratories, operators, instruments, and real-time PCR platforms will be important to establish reproducibility, robustness, transferability, and broader applicability under routine food safety testing conditions.

## 7. Conclusions

The GenoPATHX™ multiplex qPCR assay demonstrated robust analytical sensitivity, quantitative reliability, and operational robustness across the poultry-associated matrices evaluated in this study. The assay successfully integrated multiplex serovar-specific detection, direct quantification, and same-day molecular workflow within a single platform while maintaining stable amplification performance under multiplex conditions. The ability to generate quantitative and serovar-level detection data without prolonged enrichment offers a potential advantage over conventional culture-dependent or presence/absence-based approaches for *Salmonella* surveillance.

Although matrix-associated amplification variability and the influence of enrichment duration remain important considerations for practical implementations, the direct molecular workflow demonstrated favorable analytical and preliminary diagnostic performance across the evaluated poultry environmental and food-associated matrices. Collectively, these findings support the potential application of the GenoPATHX™ multiplex qPCR assay as a rapid molecular surveillance and quantitative detection platform for poultry-associated *Salmonella* monitoring. Further validation using larger multi-site studies, geographically diverse isolate collections, and inter-laboratory evaluations will help establish its broader applicability for routine food safety surveillance and regulatory testing.

## Figures and Tables

**Table 1:** Amplification Efficiency and Quantitative Performance of the GenoPATHX™ Multiplex qPCR Assay Across Singleplex and Multiplex Formats.

Format	Target	Fluorophore	Slope	Intercept	R^2^	Efficiency (%)	95% CI	Levels	Replicates	Mean SD	Mean CV (%)	Mean log10 Error	SD log10 Error	Slope *p*-Value
CKPI	*S.* Enteritidis	ROX	−3.44	40.55	0.999	95.3	93.2–97.5	7	21	0.140	0.45	0.048	0.049	0.392
CKPI	Monophasic *S.* Typhimurium	FAM	−3.51	39.59	0.997	92.9	89.4–96.6	7	20	0.207	0.80	0.074	0.084	0.714
CKPI	*S.* Typhimurium	Cy5.5	−3.32	39.21	0.994	100.1	95.6–105.1	8	23	0.154	0.52	0.106	0.131	0.403
CKPI	*S. enterica* spp.	Cy5	−3.32	38.65	0.995	99.9	95.7–104.5	8	23	0.194	0.64	0.097	0.124	0.051
Singleplex	*S.* Enteritidis	ROX	−3.41	40.03	0.999	96.5	94.5–98.7	7	21	0.132	0.44	0.043	0.051	0.392
Singleplex	*S.* Hadar	FAM	−3.22	38.46	0.996	104.3	99.7–109.3	7	20	0.326	1.12	0.085	0.099	0.234
Singleplex	Monophasic *S.* Typhimurium	FAM	−3.53	39.68	0.998	92.1	89.6–94.7	7	20	0.258	0.89	0.061	0.052	0.714
Singleplex	*S.* Muenchen	ROX	−3.28	41.02	0.998	101.8	98.2–105.7	6	17	0.238	1.00	0.061	0.059	0.504
Singleplex	*S.* Typhimurium	Cy5.5	−3.40	40.38	1.000	97.0	95.7–98.3	6	18	0.084	0.32	0.026	0.018	0.403
Singleplex	*S. enterica* spp.	Cy5	−3.21	39.30	0.987	104.9	97.7–113.1	8	23	0.407	1.23	0.150	0.211	0.051
TKPI	*S.* Hadar	FAM	−3.31	39.09	0.996	100.5	96.4–105.1	7	20	0.344	1.44	0.088	0.086	0.234
TKPI	*S.* Muenchen	ROX	−3.33	41.91	0.994	99.6	94.8–104.8	7	21	0.366	1.34	0.109	0.106	0.504
TKPI	*S.* Typhimurium	Cy5.5	−3.37	40.65	0.999	97.9	96.1–99.7	7	21	0.131	0.45	0.036	0.044	0.403
TKPI	*S. enterica* spp.	Cy5	−3.43	40.20	0.998	95.6	93.2–98.2	7	21	0.189	0.68	0.060	0.054	0.051

**Table 2:** Efficiency-Corrected Amplification Performance Determined Using LinRegPCR.

Target	Mean PCR Efficiency (E)	R^2^ Range
*S.* Typhimurium	2.0	0.995–1.000
*S.* Enteritidis	1.98	0.996–1.000
Monophasic *S.* Typhimurium	2.1	0.995–0.999
*S.* Muenchen	2.1	0.996–1.000
*S.* Hadar	2.2	0.993–1.000
*S*. *enterica* spp.	1.9	0.996–1.000

Efficiency-corrected amplification performance was determined using LinRegPCR analysis of individual amplification curves. Mean PCR efficiency values approaching 2.0 indicate near-optimal amplification kinetics.

**Table 3:** Target-specific Limits of Quantification (LoQ) and Quantitative Precision of the GenoPATHX™ multiplex qPCR Assay.

Target	Fluorophore	LoQ (GE/Reaction)	Log_10_ LoQ	Detection Rate (95% CI)	Mean Cq ± (SD)	CV (%)
*S.* Enteritidis	ROX	43.8	1.64	1.00 (0.86–1.00)	33.47 (1.20)	3.59
*S.* Hadar	FAM	219	2.34	1.00 (0.86–1.00)	32.63 (2.82)	8.65
Monophasic *S.* Typhimurium	FAM	219	2.34	1.00 (0.86–1.00)	34.29 (3.21)	9.36
*S.* Muenchen	ROX	219	2.34	1.00 (0.86–1.00)	33.09 (1.82)	5.50
*S. enterica* spp.	Cy5	219	2.34	1.00 (0.86–1.00)	34.22 (1.07)	3.13
*S.* Typhimurium	Cy5-5	43.8	1.64	1.00 (0.86–1.00)	35.68 (0.83)	2.34

**Table 4:** Target-specific Limits of Detection (LoD_95_) of the GenoPATHX™ Multiplex qPCR assay.

Target	Fluorophore	LoD_95_ (GE/Reaction)	Log_10_ LoD_95_	95% CI (GE/Reaction)	Probit Slope	Detection Model R^2^
*S.* Enteritidis	ROX	71.2	1.85	49.6–121	1.92	0.998
*S.* Hadar	FAM	168	2.23	108–345	1.71	0.994
Monophasic *S.* Typhimurium	FAM	545	2.74	271–2840	1.18	0.987
*S.* Muenchen	ROX	116	2.06	74.4–226	1.83	0.996
*S. enterica* spp.	Cy5	118	2.07	75.6–232	1.79	0.995
*S.* Typhimurium	Cy5.5	60.0	1.78	42.2–95.5	1.96	0.999

LoD95 represents the concentration predicted to yield positive amplification in 95% of replicate reactions based on probit regression analysis. Confidence intervals (CIs) represent exact 95% prediction intervals derived from fitted detection models.

**Table 5:** Repeatability and Intermediate Precision of the GenoPATHX™ Multiplex qPCR Assay Across Evaluated Concentration Levels.

Target	GE/Reaction	Mean Cq ± SD	Repeatability CV (%)	Intermediate Precision CV (%)
*S.* Enteritidis	5470	26.11 ± 0.28	1.07	1.19
	1094	29.42 ± 0.39	1.33	1.58
	**43.8**	33.47 ± 1.20	3.59	4.12
	8.75	36.88 ± 2.14	5.81	6.32
	1.75	38.94 ± 3.47	8.91	9.18
	0.35	39.87 ± 4.18	10.48	11.02
*S*. Hadar	5470	25.88 ± 0.31	1.21	1.44
	1094	29.16 ± 0.42	1.44	1.73
	**219**	32.63 ± 2.82	8.65	8.91
	43.8	36.29 ± 3.48	9.84	10.21
	8.75	38.41 ± 4.15	11.08	11.72
	1.75	39.54 ± 4.62	12.44	13.03
Monophasic *S.* Typhimurium	5470	26.34 ± 0.36	1.36	1.54
	1094	30.02 ± 0.54	1.81	2.07
	**219**	34.29 ± 3.21	9.36	9.36
	43.8	37.11 ± 4.04	10.89	11.41
	8.75	39.06 ± 4.72	12.08	12.84
	1.75	39.88 ± 5.10	13.21	14.07
*S.* Muenchen	5470	25.77 ± 0.29	1.14	1.38
	1094	29.85 ± 0.39	1.31	1.58
	**219**	33.09 ± 1.82	5.50	5.94
	43.8	36.12 ± 2.91	7.82	8.16
	8.75	38.07 ± 3.66	9.61	10.03
	1.75	39.21 ± 4.25	10.84	11.42
*S. enterica*	5470	25.94 ± 0.34	1.28	1.42
	1094	29.67 ± 0.66	2.21	2.48
	**219**	34.22 ± 1.07	3.13	3.47
	43.8	36.95 ± 1.84	4.98	5.21
	8.75	38.88 ± 2.71	6.97	7.44
	1.75	39.62 ± 3.52	8.89	9.27
*S.* Typhimurium	5470	25.52 ± 0.27	1.07	1.19
	1094	28.88 ± 0.31	1.09	1.24
	**43.8**	35.68 ± 0.83	2.34	2.69
	8.75	37.92 ± 1.47	3.87	4.12
	1.75	39.11 ± 2.54	6.49	6.93
	0.35	39.96 ± 3.92	9.81	10.28

Repeatability represents intra-assay precision evaluated under identical experimental conditions, whereas intermediate precision represents precision across independent experimental runs performed on different days within the same laboratory across independent experimental runs. Precision was assessed using coefficient of variation (CV) values derived from replicate Cq measurements across serial concentration levels. Bolded concentrations represent experimentally established limits of quantification (LoQ) for each target. Increased variability at concentrations below the LoQ is consistent with expected stochastic amplification behavior near the lower quantitative range of qPCR assays.

**Table 6:** Analytical specificity of the GenoPATHX™ multiplex qPCR assay based on inclusivity and exclusivity testing across 128 bacterial isolates.

Assay Target	Inclusivity (n/N)	Inclusivity (%)	Inclusivity 95% CI	Exclusivity (n/N)	Exclusivity (%)	Exclusivity 95% CI
Enteritidis	1/1	100.0	2.5–100.0	119/127	93.7	88.0–97.2
*S. enterica*	98/98	100.0	96.3–100.0	30/30	100.0	88.4–100.0
Hadar	1/1	100.0	2.5–100.0	124/127	97.6	93.3–99.5
Monophasic *S.* Typhimurium 1,4,[5],12:i:-	3/3	100.0	29.2–100.0	122/125	97.6	93.2–99.5
Muenchen	3/3	100.0	29.2–100.0	118/125	94.4	88.8–97.7
Typhimurium	1/1	100.0	2.5–100.0	121/127	95.3	90.0–98.3

Inclusivity was defined as correct amplification of intended target isolates, whereas exclusivity was defined as absence of amplification among evaluated non-target isolates. Confidence intervals (CIs) were calculated using the exact binomial (Clopper–Pearson) method.

**Table 7:** Effect of annealing temperature on amplification performance of the GenoPATHX™ multiplex qPCR assay.

Annealing Temperature (°C)	n	Mean Cq ± SD	CV (%)	ΔCq vs. 64 °C	95% CI	Tukey-Adjusted *p*-Value
63	72	30.58 ± 2.01	6.56	−0.23	[−0.56, 0.10]	0.126
64	72	30.81 ± 1.97	6.40	Reference	—	—
65	72	31.15 ± 2.07	6.65	+0.34	[0.07, 0.61]	0.013

Annealing temperatures of 63 °C, 64 °C, and 65 °C were evaluated across six assay targets and four fluorophore channels (n = 216 reactions). ΔCq values were estimated relative to the reference annealing temperature of 64 °C using linear mixed-effects modeling. Confidence intervals (CIs) represent Tukey-adjusted estimated marginal mean comparisons. Although a statistically significant increase in Cq was observed at 65 °C, all model-estimated shifts remained below one amplification cycle.

**Table 8:** Effect of DNA template input volume on amplification performance of the GenoPATHX™ multiplex qPCR assay.

DNA Input Volume (µL)	Mean Cq ± SD	CV (%)	ΔCq vs. 3 µL	95% CI	*p*-Value
2	33.22 ± 0.89	2.68	+0.44	0.08 to 0.80	0.018
3	32.87 ± 1.01	3.08	Reference	—	—
4	32.56 ± 1.18	3.63	−0.22	−0.58 to 0.14	0.398

DNA input volumes of 2 µL, 3 µL, and 4 µL were evaluated across CKPI and TKPI multiplex panels (n = 72 reactions). ΔCq values were estimated relative to the standard input volume of 3 µL using linear mixed-effects modeling. Confidence intervals (CIs) represent Tukey-adjusted estimated marginal mean comparisons. Although statistically significant differences were identified across input volumes, all model-estimated Cq shifts remained below one amplification cycle.

**Table 9:** Linear regression performance of the GenoPATHX™ multiplex qPCR assay in poultry matrices.

Matrix	Panel	Target	Linear Range (CFU/mL)	Slope	R^2^	Efficiency (%)	*p* Value
Chicken rinsate	CKPI	*S.* Enteritidis	3.0 × 10^7^–7.68 × 10^1^	−4.41	0.987	68.7	<0.001
Chicken rinsate	CKPI	*S.* Monophasic	3.0 × 10^7^–7.68 × 10^1^	−3.60	0.974	89.5	<0.001
Chicken rinsate	CKPI	*S.* Typhimurium	3.0 × 10^7^–7.68 × 10^1^	−3.94	0.995	79.5	<0.001
Chicken rinsate	CKPI	*S. enterica* spp.	3.0 × 10^7^–7.68 × 10^1^	−3.99	0.957	78.0	<0.001
Ground turkey	TKPI	*S.* Hadar	3.0 × 10^7^–7.68 × 10^1^	−3.57	0.906	90.6	<0.001
Ground turkey	TKPI	*S.* Muenchen	3.0 × 10^7^–7.68 × 10^1^	−2.90	0.844	121.0	<0.001
Ground turkey	TKPI	*S.* Typhimurium	3.0 × 10^7^–7.68 × 10^1^	−2.80	0.960	128.0	<0.001
Ground turkey	TKPI	*S. enterica* spp.	3.0 × 10^7^–7.68 × 10^1^	−3.45	0.883	95.1	<0.001

Artificially inoculated chicken rinsate and ground turkey matrices were evaluated using five-fold serial dilutions with direct DNA extraction and multiplex qPCR analysis without enrichment. Linear regression analysis was performed by plotting Cq values against log10 CFU/mL. Amplification efficiency was calculated from regression slope values. All regression models were statistically significant (*p* < 0.001).

**Table 10:** Lowest Reproducibly Detected Concentrations of the GenoPATHX™ multiplex qPCR assay in Artificially inoculated matrices.

Matrix	Panel	Target	Lowest Reproducibly Detected Concentration (CFU/mL)	Detection Frequency (%)
Chicken rinsate	CKPI	*S.* Enteritidis	7.68 × 10^1^	100
Chicken rinsate	CKPI	Monophasic *S.* Typhimurium	7.68 × 10^1^	100
Chicken rinsate	CKPI	*S.* Typhimurium	7.68 × 10^1^	100
Chicken rinsate	CKPI	*S. enterica*	7.68 × 10^1^	100
Ground turkey	TKPI	*S.* Hadar	7.68 × 10^1^	100
Ground turkey	TKPI	*S.* Muenchen	7.68 × 10^1^	100
Ground turkey	TKPI	*S.* Typhimurium	7.68 × 10^1^	100
Ground turkey	TKPI	*S. enterica*	7.68 × 10^1^	100
Ground beef	TKPI	*S.* Hadar	3.84 × 10^2^	100
Ground beef	TKPI	*S.* Muenchen	3.84 × 10^2^	100
Ground beef	TKPI	*S.* Typhimurium	3.84 × 10^2^	100
Ground beef	TKPI	*S. enterica*	3.84 × 10^2^	100

Lowest reproducibly detected concentrations were defined as the lowest bacterial concentrations consistently detected across replicate reactions within each evaluated matrix. Detection frequencies were determined using multiplex qPCR following direct DNA extraction without enrichment.

**Table 11:** Detection Frequency, Mean Colony Count, and Amplification Performance of the GenoPATHX™ Multiplex qPCR Assay in Directly Inoculated Ground Beef.

Panel	Dilution	Mean Colony Count ± SD	Positive Replicates (*n*/*N*)	Mean Cq ± SD
CKPI	−4	86.3 ± 74.8	3/3	34.99 ± 0.65
CKPI	−5	3.3 ± 3.2	2/3	36.21 ± 1.08
CKPI	−6	0.0 ± 0.0	1/3	37.13
CKPI	−7	0.0 ± 0.0	1/3	37.39
TKPI	−4	522.7 ± 279.5	3/3	36.16 ± 0.63
TKPI	−5	9.7 ± 4.5	3/3	36.60 ± 1.24
TKPI	−6	1.7 ± 1.2	2/3	36.86 ± 0.23
TKPI	−7	0.0 ± 0.0	0/3	ND

Ground beef samples were directly inoculated with serially diluted mixed-serovar suspensions and analyzed using multiplex qPCR following direct DNA extraction without enrichment. Mean colony counts were determined by triplicate plate enumeration and are presented as mean ± standard deviation (SD). Detection frequency is reported as the number of positive replicates relative to the total number of reactions performed. Mean Cq values are presented for positive qPCR replicates only. ND, no detectable amplification.

**Table 12:** Matrix effect evaluation of GenoPATHX™ multiplex qPCR standard curves in PBS and chicken rinsate.

KPI Panel	Target	Slope (PBS)	Slope (Chicken Rinsate)	Efficiency % (PBS)	Efficiency % (Chicken Rinsate)	Mean ΔCq (Chicken − PBS)	95% CI	Adjusted *p*-Value (Slope)	Adjusted *p*-Value (ΔCq)
CKPI	Enteritidis	−3.70	−3.81	86.3	82.9	−0.51	−0.61 to −0.40	0.029	5.96 × 10^−11^
CKPI	*S. enterica*	−3.66	−3.83	87.6	82.5	−0.78	−0.91 to −0.64	0.013	1.10 × 10^−12^
CKPI	Monophasic *S.* Typhimurium	−3.77	−3.84	84.3	82.3	−0.94	−1.11 to −0.77	0.359	5.96 × 10^−11^
CKPI	Typhimurium	−3.72	−3.87	85.7	81.4	−1.12	−1.27 to −0.98	0.029	8.77 × 10^−16^
TKPI	*S. enterica* spp.	−3.72	−3.79	85.7	83.6	−0.88	−1.05 to −0.71	0.359	5.08 × 10^−12^
TKPI	Hadar	−3.49	−3.80	93.6	83.3	−0.93	−1.14 to −0.71	0.001	3.83 × 10^−09^
TKPI	Muenchen	−3.72	−3.82	85.9	82.7	−1.64	−1.84 to −1.44	0.297	3.25 × 10^−16^
TKPI	Typhimurium	−3.55	−3.67	91.5	87.2	−1.43	−1.61 to −1.25	0.151	1.97 × 10^−16^

Comparative standard curve analyses were performed using five-fold serial dilutions prepared in phosphate-buffered saline (PBS) and chicken rinsate matrices. Amplification efficiency was calculated from regression slope values. Mean ΔCq values represent matrix-associated shifts relative to PBS. Confidence intervals (CIs) correspond to estimated marginal mean differences derived from generalized least squares modeling. Adjusted *p*-values were corrected for multiple comparisons using false discovery rate (FDR) adjustment.

**Table 13:** Diagnostic performance of the GenoPATHX™ *S. enterica* spp. Assay relative to the USDA-FSIS Reference Culture method.

Metric	GenoPATHX™ *S. enterica* Direct Workflow	GenoPATHX™ *S. enterica* 3 h Actero™ Workflow
True positives (TP)	17	10
False positives (FP)	2	5
False negatives (FN)	4	11
True negatives (TN)	21	18
Total samples (N)	44	44
Sensitivity, % (95% CI)	81.0 (58.1–94.6)	47.6 (25.7–70.2)
Specificity, % (95% CI)	91.3 (72.0–98.9)	78.3 (56.3–92.5)
Positive predictive value (PPV), % (95% CI)	89.5 (66.9–98.7)	66.7 (38.4–88.2)
Negative predictive value (NPV), % (95% CI)	84.0 (63.9–95.5)	62.1 (42.3–79.3)
Accuracy, % (95% CI)	86.4 (72.6–94.8)	63.6 (47.8–77.6)
Prevalence, %	47.7	47.7
Cohen’s kappa (κ)	0.726	0.26
Accuracy *p*-value (vs. NIR)	1.96 × 10^−6^	0.0866
McNemar’s χ^2^	0.17	1.56
McNemar’s *p*-value	0.683	0.211

Diagnostic performance metrics were calculated relative to the USDA-FSIS reference culture method using 44 poultry environmental samples. Confidence intervals (CIs) were calculated using the exact binomial method. NIR, no-information rate. Cohen’s kappa (κ) values were interpreted according to standard agreement classification criteria.

**Table 14:** Confusion Matrices Comparing GenoPATHX™ Assay Workflows with the USDA-FSIS Reference Culture Method for Detection of *Salmonella* in Poultry Environmental Samples.

Workflow	GenoPATHX™ Result	Culture Positive	Culture Negative
*S. enterica* Direct Workflow	Positive	17	2
	Negative	4	21
3 h Actero™ Enrichment Workflow	Positive	10	5
	Negative	11	18

Confusion matrices summarize concordance between GenoPATHX™ *S. enterica* qPCR detection workflows and the USDA-FSIS reference culture method for *Salmonella* detection in poultry environmental samples (n = 44).

## Data Availability

Data are contained within the article and supplementary materials.

## Supplementary Materials

The following supporting information can be downloaded at: https://www.mdpi.com/article/10.3390/pathogens15080791/s1, Table S1: Amplification Efficiency Analysis; Table S2: Limit of Detection (LoD95), Limit of Quantification (LoQ), and Precision; Table S3: Analytical Inclusivity and Exclusivity; Table S4: Matrix Effect Evaluation; Table S5: Short Enrichment Study; Table S6: Diagnostic Validation; Table S7: Whole-Genome Sequencing Results.
